# The complete local genotype–phenotype landscape for the alternative splicing of a human exon

**DOI:** 10.1038/ncomms11558

**Published:** 2016-05-10

**Authors:** Philippe Julien, Belén Miñana, Pablo Baeza-Centurion, Juan Valcárcel, Ben Lehner

**Affiliations:** 1 EMBL/CRG Systems Biology Research Unit, Centre for Genomic Regulation (CRG), The Barcelona Institute of Science and Technology, Dr Aiguader 88, Barcelona 08003, Spain; 2 Universitat Pompeu Fabra (UPF), Barcelona 08003, Spain; 3 Gene Regulation, Stem Cells and Cancer Program, Centre for Genomic Regulation (CRG), The Barcelona Institute of Science and Technology, Dr Aiguader 88, Barcelona 08003, Spain; 4 Institució Catalana de Recerca i Estudis Avançats (ICREA), Pg. Lluís Companys 23, 08010 Barcelona, Spain

## Abstract

The properties of genotype–phenotype landscapes are crucial for understanding evolution but are not characterized for most traits. Here, we present a >95% complete local landscape for a defined molecular function—the alternative splicing of a human exon (FAS/CD95 exon 6, involved in the control of apoptosis). The landscape provides important mechanistic insights, revealing that regulatory information is dispersed throughout nearly every nucleotide in an exon, that the exon is more robust to the effects of mutations than its immediate neighbours in genotype space, and that high mutation sensitivity (evolvability) will drive the rapid divergence of alternative splicing between species unless it is constrained by selection. Moreover, the extensive epistasis in the landscape predicts that exonic regulatory sequences may diverge between species even when exon inclusion levels are functionally important and conserved by selection.

## Abstract (web summary)

Genotype–phenotype landscapes are an important characteristic for understanding the evolution of traits. Here the authors construct the local landscape for the alternative splicing of FAS/CD95 exon 6, revealing the regulation of splicing and the evolution of regulatory information between species.

## Graphical abstract

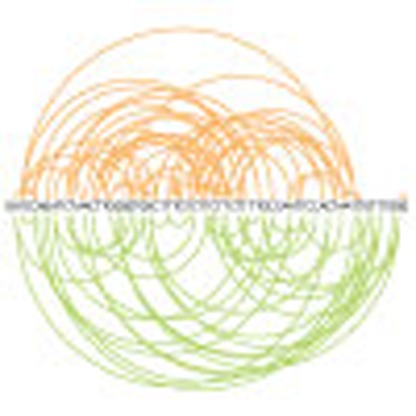

Genotype–phenotype landscapes describe the mapping between changes in DNA sequence and the changes in phenotypic traits. Despite their centrality to many areas of biology, including human disease genetics and both molecular and phenotypic evolution, they are poorly characterized. One reason for this is their vast combinatorial size^1^.

Alternative splicing of messenger RNA (mRNA) precursors is an important regulatory step in gene expression that is perturbed in multiple diseases including cancer^2^. Alternatively spliced exons are often relatively short, making the goal of accurately quantifying the effects of all possible single and double mutations on regulation—and so a complete description of the local genotype–phenotype landscape for a step in gene expression—a realistic possibility.

Alternatively spliced transcripts are proposed to contain four classes of regulatory elements that promote and repress their inclusion into a mature transcript, by binding to *trans* factors: exonic splicing enhancers, exonic splicing silencers^2^,^3^, intronic splicing enhancers and intronic splicing silencers^2^,^3^. Mutations in exonic splicing regulatory sequences are known to cause a variety of human diseases^4^. Several recent studies have systematically analysed the evolution of alternative splicing, concluding that inclusion levels are poorly conserved between species for most alternatively spliced exons^5^,^6^,^7^.

To better understand how alternative splicing is regulated, affected by mutations, and evolves, we have comprehensively quantified the effects of nearly all single and double mutations in the 63 nucleotides of the death receptor FAS/CD95 exon 6, whose alternative splicing generates isoforms with opposite roles in apoptosis^8^,^9^. The >95% complete local genotype–phenotype landscape reveals that single mutations at >90% of positions and >60% of all single nucleotide variants alter splicing, demonstrating a rich and widespread landscape of splicing regulatory sequences beyond the classical organization into discrete ‘enhancer' and ‘silencer' elements. Contrasting effects are frequently observed for mutations in adjacent nucleotides and for different nucleotide substitutions, with single mutations altering inclusion to nearly any quantitative level. Analysis of double mutations reveals that the wild-type (wt) exon is a local peak of mutational robustness and that the genotype–phenotype landscape is highly epistatic, for example with 74% of mutations with no effect on splicing alone modifying the outcome of another mutation in the exon.

Thus, despite indicating selection on both inclusion and robustness, the landscape reveals that alternative splicing is very sensitive to mutation and will diverge rapidly during evolution unless constrained by purifying selection. Moreover, the extensive epistasis in the landscape means that selection on inclusion levels alone may still result in the rapid divergence of splicing regulatory sequences between species. Thus, even when function (percent spliced in) is maintained, the exonic regulatory sequences responsible for this function may still diverge between species.

## Results

### A comprehensive splicing genotype-to-phenotype map

**Figure 1:**
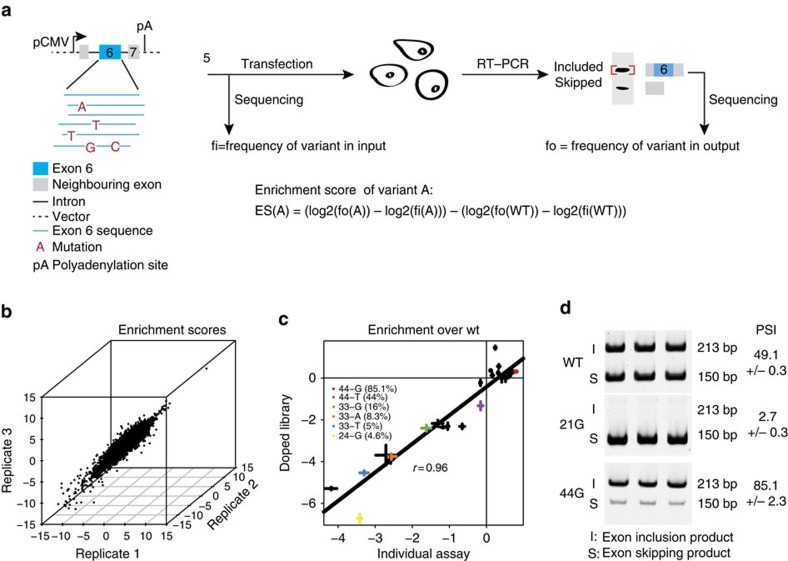
Massively parallel analysis of the effects of all exonic mutations on Fas exon 6 inclusion. (**a**) Overview of the experimental procedure. (**b**) Correlation between the enrichment scores over wt of the three biological replicates for one and two mutations variants. The Pearson correlation coefficients range between 0.88 and 0.91. (**c**) Correlation between enrichment scores for 25 variants determined in transfection assays of individual mutant clones and by massively parallel RNA analysis of the transfected library products. Error bars show standard deviation. For reference, the PSI is shown for some variants. (**d**) Examples of variants' inclusion scores determined by individual transfection assays in triplicate.

We generated a library of sequence variants designed to cover all possible single and double mutations in exon 6 of human FAS/CD95 using doped oligonucleotide synthesis^10^. The library was cloned into a minigene cassette spanning FAS exons 5–7 (which does not support protein expression), transfected into HEK293 cells under conditions that lead to approximately 50% exon inclusion, matching the levels of exon 6 inclusion in endogenous transcripts in this cell line, RNA was isolated and the inclusion score of each variant exon was quantified by reverse transcription, PCR amplification with minigene-specific primers and deep sequencing (Fig. 1a). An enrichment score for each variant was determined by comparing the inclusion score in the spliced RNA over the frequency of the variant in the input library (Fig. 1a).

**Figure 2:**
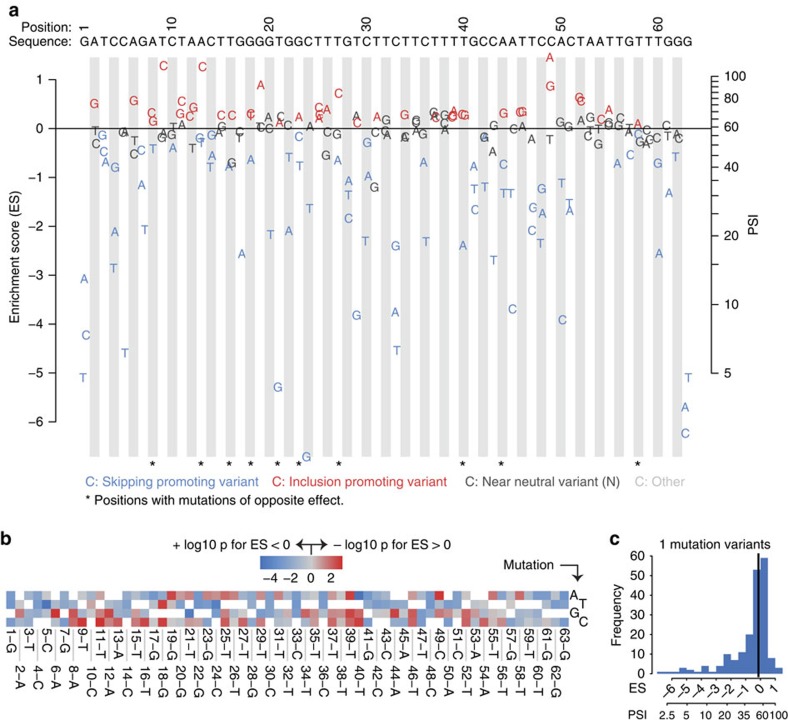
Effects of all possible single mutations on alternative splicing of Fas exon 6. High mutational plasticity of alternative splicing. (**a**) Dotchart of the enrichment scores for all 189 single mutation variants. Colour code indicates whether the variant's effect is statistically different from that of WT sequence (red indicates increased inclusion, blue increased skipping). (**b**) Heatmap displaying the extent of statistical difference from wt sequence for all 189 variants. Colour scale represents the log10 of the *P* value multiplied by 1 or −1 for variants with negative and positive enrichment scores, respectively. (**c**) Distribution of enrichment scores for all single mutants. The vertical black line represents the median of the distribution. PSI values were estimated from the enrichment scores using the relationship shown in Supplementary Fig. 3B.

The enrichment inclusion scores were highly correlated across three replicates (Pearson correlation coefficient, *r* from 0.88 to 0.91, Fig. 1b) and also highly correlated to the inclusion enrichment scores measured for 25 exon variants assayed and quantified individually (Pearson *r*=0.96, Fig. 1c,d; Supplementary Figs 1 and 9). The correlation was maintained when testing these 25 individual mutations in a second cell type displaying a higher (95%) basal level of inclusion (HeLa, Supplementary Figs 2, 3A and 9), suggesting largely consistent effects across different cell types. A small number of mutations had enrichment scores predicting a Percent Spliced In (PSI) >100% (Fig. 2a,c and Supplementary Fig. 3) suggesting that they might have effects on gene expression beyond changes in splicing. However the strong correlation between enrichment scores and PSI values across the individually assayed mutants (Fig. 1b and Supplementary Fig. 3) indicates that this is rare.

### Effects of all single mutations on exon inclusion

Out of the 189 possible single mutations, 74 (39%) significantly reduced exon inclusion, 41 (22%) promoted inclusion and 73 (39%) had no statistically distinguishable effect on splicing (Welch's unequal variances *t*-test, false discovery rate (FDR)<0.05, Fig. 2a,b and Supplementary Data 1). The distributions of mutation effects were similar for synonymous and non-synonymous mutations (Supplementary Fig. 4A; Mann–Whitney *U* test *P*=0.82; 49 synonymous and 140 non-synonymous mutations). In total, single nucleotide changes at 58/63 positions (92%) affected splicing, demonstrating that splicing regulatory sequences are distributed across nearly every nucleotide in the exon (Fig. 2a), expanding previous estimates^3^,^11^,^12^.

While the results partially recapitulate the location of exonic splicing enhancers and silencers coarsely defined through previous deletion/substitution of exonic regions^13^,^14^,^15^ (Supplementary Fig. 4B), our nucleotide level resolution analyses reveal a much richer regulatory content, as illustrated by the distinct effects of adjacent nucleotides and of alternative nucleotide substitutions of the same residue (Fig. 2a). For example, while replacement of the central silencer—composed of tandem TTCT PTB consensus motifs—by a variety of sequences led to higher levels of exon inclusion^13^, multiple individual mutations (in particular at C positions) in these motifs lead to exon skipping (Fig. 2a and Supplementary Fig. 4B). Indeed, different nucleotide substitutions at 9 (14%) positions scattered through the exon lead to opposite effects on inclusion/skipping (Fig. 2a).

The distribution of mutation effects (Fig. 2c) is shifted towards reduced inclusion (one sample Wilcoxon test *P*<10^−6^), indicating selection for sequences that promote inclusion in the wt exon. However, single nucleotide changes can quantitatively alter splicing over nearly the complete range of inclusion levels (Fig. 2a,c and Supplementary Fig. 3). This high mutation sensitivity means that the alternative splicing of this exon will evolve extremely rapidly unless constrained by purifying selection.

### Analysis of double mutations

**Figure 3:**
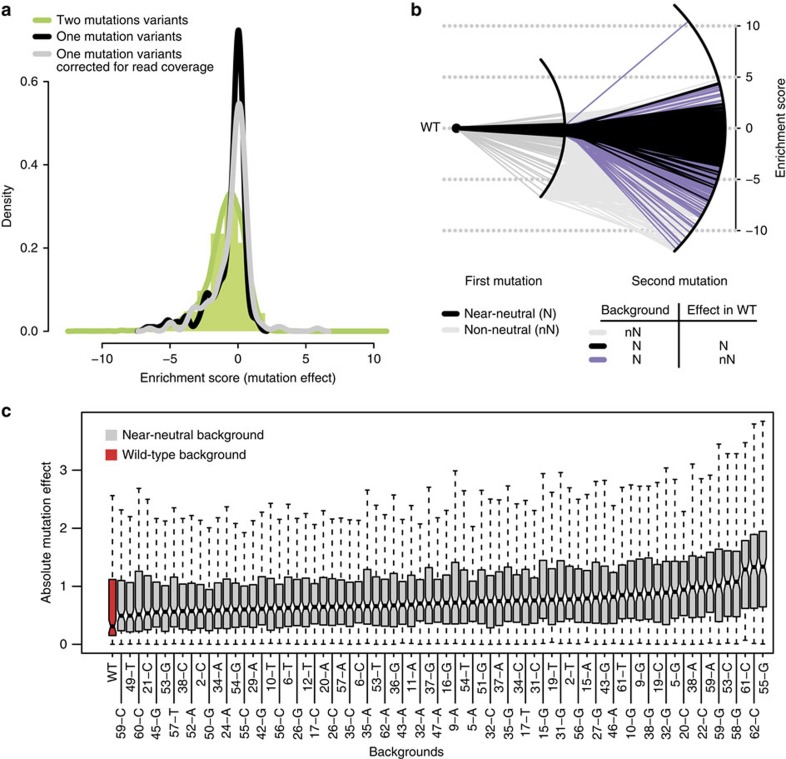
Effects of double mutations on FAS exon 6 inclusion. The wt sequence is more robust to the effects of mutations than its immediate neighbours in genotype space with the same exon inclusion. (**a**) Distribution of enrichment scores of 16,278 double mutants (green histogram and green curve). The distribution of the enrichment scores of single mutants without (black curve) or with (grey curve) correction for differential sequencing coverage is shown for comparison. (**b**) Representation of the near-neutral network. Left side: black lines show near-neutral mutations in the wt background. Right side: grey lines derive from non-neutral backgrounds. Black and purple lines derive from near-neutral backgrounds and are near-neutral (black) or non-neutral (purple) in WT. (**c**) Absolute mutation effect of all variants in the wt (red) and in each of the 73 near-neutral backgrounds (grey).

In addition to all single nucleotide changes, the library also allowed us to quantify the effects on inclusion of 95% (16,728/17,577) of double mutants (Supplementary Data 2). We first compared the distribution of mutation effects of single and double mutants and observed that, although a similar range of inclusion is observed for both sets, double mutants have a broader distribution of mutation effects, even when differential sequencing coverage is accounted for (Fig. 3a).

We next compared the effects of all single mutations in the wt exon to their effects in exons that differ from the wt by one nucleotide but maintain the same level of inclusion (we term these immediate neighbours in genotype space as ‘near-neutral' with respect to their effects on exon inclusion, Fig. 3b). Surprisingly, this revealed that mutations have a smaller effect on splicing in the wt sequence than in the immediate neighbours in genotype space displaying the same level of inclusion; the median absolute effect of a mutation was smaller for the wt exon than for all of the 73 near-neutral neighbours (Fig. 3c). The interquartile range of relative mutation effects was also narrower in the wt than in the near-neutral backgrounds (Supplementary Fig. 5A), even when differential sequencing coverage is accounted for (Supplementary Fig. 5B). Thus, the wt exon is more robust to the effects of mutations than closely related sequences with the same level of inclusion, indicating that the wt exon is located close to a local peak of mutational robustness in genotype space.

### Epistasis and the evolution of splicing

We quantified epistasis—the unexpected outcome of combining two mutations—using an empirical epistasis score (Supplementary Fig. 6). We analysed the interactions of near-neutral mutations, as they are crucial for understanding molecular evolution^16^,^17^. Focusing on these mutations also reduces the multiple testing burden, increasing statistical power.

**Figure 4:**
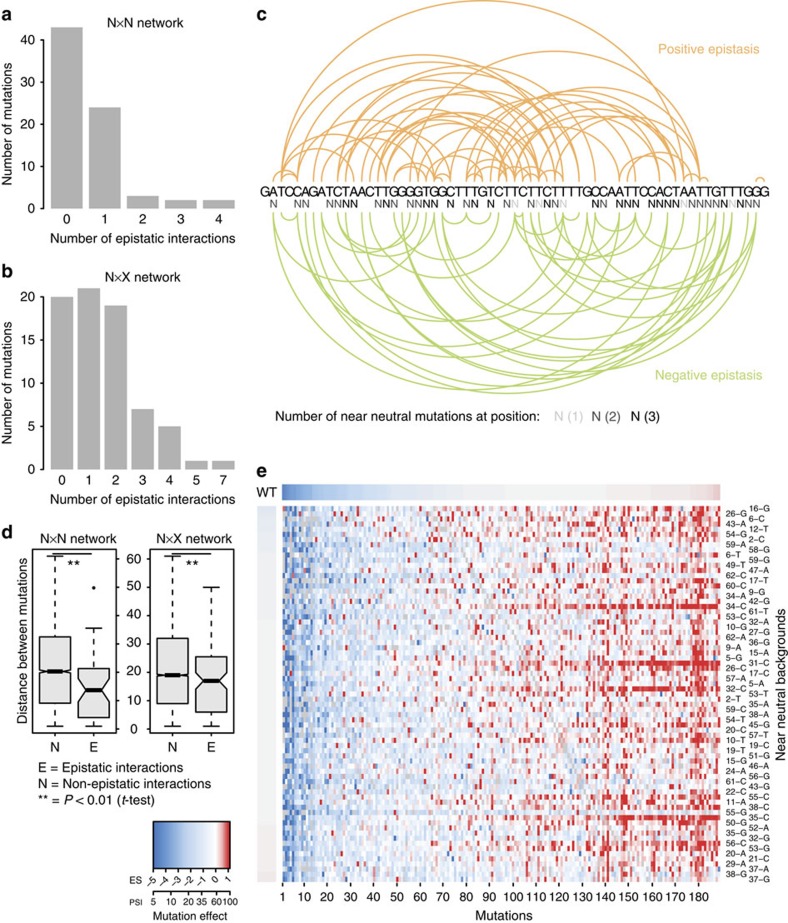
Epistasis and the evolution of alternative splicing. (**a**,**b**) Distribution of the number of epistatic interactions with near-neutral mutations for near-neutral (A, N × N network) or all (B, N × X network) mutations. (**c**) Visualization of positive (orange) and negative (green) epistatic interactions between near-neutral mutations and any other mutation. The number of near-neutral mutations at each site is depicted by different shades of grey (light=1 near-neutral mutation at the site; intermediate=2; dark=3). (**d**) Linear distance between epistatically and non-epistatically interacting pairs of mutations for the N × N and N × X networks. (**e**) Effect of all single mutations (columns) in near-neutral backgrounds (rows). Rows and columns are ordered by the effect of the mutation in the wt background (as represented by the horizontal and vertical scales).

In the complete dataset, 54/189 mutations that alter splicing alone (29%) and 31/73 mutations that do not (42%) interact epistatically (FDR<0.05; see Methods) with at least one of the 73 near-neutral mutations (Fig. 4a,b). In total, 40% (76/189) of all mutations interact with a near-neutral mutation and 74% (54/73) of near-neutral mutations modify the outcome of at least one mutation. This means that a mutation with no detectable effect on splicing at 41 of the 44 positions where such mutations can occur (93%) interacts with another site in the exon (Fig. 4c). Although the epistatic interactions occur between mutations dispersed throughout the exon (Fig. 4c), they are enriched for local interactions, suggesting more frequent compensation and synergy between mutations in local regulatory sequences (Fig. 4d).

The degree distribution of the number of interactions per mutation is long-tailed for both mutations that do (Fig. 4a) and do not (Fig. 4b) affect splicing alone, highlighting the presence of epistasis hubs, that is, mutations whose effects are highly context dependent, switching from strong to small effect depending on the genetic background (Fig. 4e and Supplementary Fig. 5C). Analysis of the quantitative epistasis scores for all mutation combinations supports these conclusions (Supplementary Fig. 7A–F). Overall, however, 52% of the total variance in splicing in the double mutants was explained by a linear model without interactions (Supplementary Fig. 6).

## Discussion

In this study we have mapped a >95% complete genotype–phenotype landscape covering two steps in nucleotide evolution for a function of a segment of human DNA. In contrast to studies that have examined the effects of a large number of sequence variants in a few short regions on the alternative splicing of an exon^3^,^18^, our aim was to systematically determine the effects of all possible individual and double mutations in all nucleotides in an exon. This allowed us to examine for the first time a near-complete local genotype–phenotype landscape for alternative splicing. The findings have implications for multiple areas of biology, including human genetics, molecular biology and evolution.

First, the landscape reveals that alternative splicing is very sensitive to mutation, with variants in >90% of exonic bases changing inclusion. It is therefore reasonable to expect that many rare variants and somatic mutations in alternatively spliced exons will alter splicing. Consistent with this, we previously found that synonymous cancer driver mutations are often associated with changes in splicing but not with other regulatory effects^19^. Taking these two results together, we propose that changed alternative splicing may be a more frequent mechanistic connection between genetic variants in exons and altered phenotypes than is currently appreciated.

Second, the complete local landscape demonstrates that the current view of splicing regulatory information as organized into discrete exonic splicing enhancers and silencers oversimplifies the wealth of regulatory information in RNA sequences. Regulatory information is dispersed throughout nearly every nucleotide in the exon in overlapping positively and negatively acting sequences. Additional mechanistic studies will be required to understand how such competitive effects of sequences and their cognate *trans*-acting factors modulate exon definition, spliceosome recruitment and activation.

Third, the high intrinsic mutation sensitivity of alternative splicing is likely to account for the rapid divergence of alternative splicing that has been observed between species^5^,^6^,^7^,^12^. Even single mutations are able to alter the inclusion of an exon to almost any level and mutations at nearly all bases alter splicing. The neutral expectation is, therefore, that alternative splicing will diverge very rapidly unless it is constrained by selection. Analysis of a smaller number of mutations in SMN1 exon 9 (ref. 12) also supports this conclusion.

Fourth, the landscape suggests that selection may be acting on the robustness of splicing regulatory mechanisms as well as on levels of inclusion: the wt exon is more robust to the effects of mutations than its immediate neighbours in genotype space with the same level of inclusion. Mutational robustness can arise as a by-product of selection for robustness to environmental or stochastic variation^20^,^21^, and we propose that this may be the case for the mutational robustness of splicing.

Fifth, the landscape helps explain why sequence conservation can be a poor indicator of functional importance in exonic regulatory sequences. The extensive epistasis in the landscape means that the accumulation of near-neutral mutations will ‘switch'^22^,^23^ the effects of nucleotide changes at other sites in the exon: mutations that previously altered splicing will no longer do so and vice versa. This will drive the divergence of regulatory information in the exon even when function is maintained.

Finally, analysis of the epistatic interaction network revealed the presence of ‘hub' mutations that interact with many other mutations in the exon. Hubs are also observed in the epistasis networks constructed by testing how mutations in different genes combine to alter viability^24^,^25^. One interpretation of the hubs is that they are mutations that have a large effect on splicing, but only in some genetic backgrounds. For example, inactivation of a particular binding site for a protein that promotes exon inclusion may only affect splicing when at least one of several other binding sites for positively acting factors is weakened. However, the precise molecular mechanisms will need to be elucidated in future work.

As for most analyses of the effects on alternative splicing of mutations in *cis* regulatory sequences, our study employed a minigene construct, an approach that has provided abundant insights into physiological and pathological splicing regulation^26^. In future work it will be important to extend this approach using mutagenesis of endogenous genomic loci, as well as to map the complete local genotype–phenotype landscapes for different biological functions and mechanisms of gene regulation. In this way it will be possible to systematically analyse how the properties of genotype–phenotype landscapes change depending on genomic context and the function and mechanism being investigated. A comprehensive empirical analysis of how molecular mechanisms constrain and facilitate the evolution of both genotypes and phenotypes should therefore be possible.

## Methods

### Doped library construction

PCR primers used in this study are illustrated in Supplementary Fig. 8 and listed in Supplementary Table 1. A 135 nucleotide-long degenerate oligonucleotide library was ordered from Trilink Biotechnologies. Library oligonucleotides include the sequence of FAS exon 6, doped at each position with 1.2% of each of the three non-reference nucleotides, flanked by invariant sequences corresponding to 22 nucleotides of the 3′ end of intron 5 and 50 nucleotides of the 5′ end of intron 6, designed for optimal Gibson cloning of the library.

5′ TGTCCAATGTTCCAACCTACAGgatccagatctaacttggggtggctttgtcttcttcttttgccaattccactaattgtttgggGTAAGTTCTTGCTTTGTTCAAACTGCAGATTGAAATAACTTGGGAAGTAG 3′, where lowercase indicates doped FAS exon 6 sequences and uppercase invariant intron sequences. The predicted composition is a library with 10% wt, 23% single mutants and 27% of double mutant sequences.

### Doped library amplification

A total of 20 ng of single-stranded doped library was amplified for 18 cycles using the flanking primers FAS_i5_GC_F/R illustrated in Supplementary Fig. 8 and listed in Supplementary Table 1 with Taq PLUS precision (600211-51, Agilent technologies) following manufacturer's instructions.

### Doped library subcloning

The amplified library was recombined with pCMV FAS wt minigene exon 5-6-7 (ref. 27) using In-Fusion HD Cloning kit (639649, Clontech) in a 1:8 vector:insert optimized ratio and transformed into Stellar competent cells (636766, Clontech) to maximize the number of individual transformants, which was around 600,000 individual clones per library. Overall 16 individual clones were Sanger-sequenced, of which 2 (13%) were found to be wt, 3 (19%) were single mutants and 3 (19%) double mutants, in reasonable accordance with the theoretical expectation. After bacterial transformation following Clontech recommendations for chemical competent cells the population of transformed bacteria was selected in LB medium in the presence of ampicillin and the plasmid library was purified using Quiagen plasmid maxi kit (50912163, Quiagen) and quantified using a Nanodrop spectrophotometer.

### Input-doped library

Overall 20 ng of the FAS-doped library was amplified using GoTaq flexi DNA polymerase (M7806, Promega) for 25 cycles with intronic primers FAS_i5_GC_F and FAS_i6_GC_R, resulting in a 135 nucleotide PCR band, which was gel-purified and Solexa-sequenced. The proportion of each variant in the library was used for enrichment score normalization.

### Cell transfection

A total of 10 ng of cloned FAS-doped library was transfected in triplicates into 300,000 HEK293 cells (ATCC CRL-1573, Mycoplasma free) in 6-well plates using Lipofectamine 2000 (11668027, Life Technologies) and OPTIMEM I reduced serum medium (31985-047, Life Technologies). Six hours post-transfection, cell culture medium was replaced by DMEM Glutamax (61965-059, Life Technologies) containing 10% FBS and Pen/Strep antibiotics (15070063, Life technologies). Forty-eight hours post transfection total RNA was purified using the automated Maxwell LEV 16 simplyRNA tissue kit (AS1280, Promega). cDNA was synthetized from 500 ng total RNA using Superscript III (18080085, Life Technologies) and 2 pmol of minigene-specific primer PT2, complementary to plasmid backbone sequences located towards the 3′ end of FAS minigene transcripts, to avoid detection of endogenous FAS RNAs. cDNAs were amplified by PCR using PT1, GTCGACGACACTTGCTCAAC and PT2, AAGCTTGCATCGAATCAGTAG primers, again corresponding to pCMV vector sequences present in Fas minigene transcripts, using GoTaq flexi DNA polymerase (M7806, Promega). PCR products were fractionated in a 2% agarose gel, the band corresponding in size to the amplification product of FAS exon 6 inclusion was excised, purified using Quiaquick Gel extraction kit (50928704, Quiagen) and quantified using a Nanodrop spectrophotometer.

One microgram each of three independent amplifications of the input library and 1 μg each, of three biological replicates of purified exon inclusion PCR products were tagged with unique 8-mer barcode sequences (Supplementary Table 1), pooled and sequenced at the EMBL Genomics Core Facility where Illumina Ampliseq PCR-free libraries were prepared and run as two different paired-end lanes on an Illumina HiSeq2000.

### Validation of selected mutants

Plasmids corresponding to 25 selected single FAS minigene mutants were obtained using Quickchange site-directed mutagenesis (210519, Agilent technologies) and primers designed with PrimerX (http://www.bioinformatics.org/primerx/). Mutants were verified by Sanger sequencing. Individual FAS mutants were transfected into Hek293 or HeLa (ATCC CCL-2, mycoplasma free) cell lines in triplicates to quantify the ratio between exon 6 inclusion and skipping^13^,^14^. Briefly, RT-PCR products were fractionated by electrophoresis in 6% polyacrylamide gels in 1 × TBE and Sybr safe staining (S33102, Life Technologies) and bands corresponding to exon inclusion or skipping quantified using ImageJ 1.47v (NIH, USA).

### Sequencing

Each library yielded between 15.7 and 39.6 millions reads for each sequencing mate. The median sequencing coverage in the input was between 12,200 and 30,788 and between 17 and 60 reads for single mutants and double mutants, respectively. The median sequencing coverage in the output was between 13,673 and 17,896 and between 18 and 28 reads for single mutants and double mutants, respectively. The wt sequence was present between 1.14 and 2.80 million times in all the libraries. This high coverage guarantees a higher confidence in measured enrichment scores.

### Computation of splicing enrichment scores

The raw sequencing files were demultiplexed using the SABRE software (https://github.com/najoshi/sabre) and paired reads from each distinct replicate were merged using PEAR^28^ with the following arguments: -m 119 -v 63 -n 119 -j 2. Reads were reverse-complemented when needed and trimmed using the seqtk trim tool (https://github.com/lh3/seqtk) with the parameters -b 26 -e 30. The number of occurrences of each variant in the library was counted using fastx_collapser (http://hannonlab.cshl.edu/fastx_toolkit/) and a custom python script.

In total the library consisted of 149,387 variants containing between 0 and 40 mutations. For each variant we computed the median frequency in the input and selection replicates libraries. The enrichment score of each variant (termed ES) was calculated as the ratio between its frequency in the selection library and in the input library. These scores were normalized by the wt score (the wt score is then 1) and log2 transformed (the wt score is then 0). Variants with more than two mutations were not considered in downstream analyses.

### Experimental evaluation of enrichment scores

We assayed and quantified the exon inclusion of 25 single mutation variants transfected individually into HEK293 cells by reverse transcription (RT)-PCR, and compared them to the enrichment scores of the same variants determined by deep sequencing in the library experiment (Fig. 1c,d and Supplementary Fig. 1).

### Converting enrichment scores into PSI values

To convert enrichment scores into PSI values, we plotted the enrichment scores of 24 single mutation variants versus their experimentally determined inclusion levels (Supplementary Fig. 3B). By fitting an exponential curve through the plotted data, it is possible to predict the PSI value of a given mutant from the value of its enrichment score. The entire mutant library has an overall enrichment score of −0.26, corresponding to a predicted PSI value of 52%; this is in good agreement with the bulk library's experimentally determined PSI value of 53%.

### Statistical tests

The statistical significance of the difference in exon inclusion of each variant was computed using a Welch's unequal variances *t*-test comparing the non-normalized enrichment of each variant in each replicate (log2 (fo(A))−log2 (fi(A))) to the enrichment of the wild-type (WT) in the same replicates (log2 (fo(WT))−log2 (fi(WT))). This approach allows the values of all generated replicates to be used in order to maximize statistical power. The *P*-values associated to each one mutation variant (*n*=189) were corrected for multiple testing (false discovery rate, Benjamini–Hochberg procedure^29^). An enrichment score was considered significantly different from wt if the FDR was below 0.05. Variants whose enrichment score did not differ from wt were classified as near-neutral unless their variance was higher than 0.15 (the variant was then not classified and labelled X). All statistical analyses were performed using the R software.

### Comparisons of mutation effect distributions

Due to the library design, the single and double mutations variants show a strong difference in sequencing coverage in the input library (median coverage is 16,275 and 25 reads and average 22,233 and 355.4 reads for single and double mutations variants, respectively). As lower reads coverage can lead to higher sampling stochasticity and thus higher variance, we corrected for read coverage when comparing distributions of mutation effects (Fig. 3a). Single mutation variants were sampled from a restricted pool of reads with the average number of reads per variant set equal to that of the double mutation reads (that is, we resampled 6,455 reads instead of the initial set of 4,202,037). The probability for a variant's read to be sampled was equal to its median frequency in the selection library. Distribution breadth was assessed using the interquartile range (third quartile minus first quartile). The distribution of mutation effects in WT was resampled 73 times (the number of near-neutral backgrounds) as described above. The interquartile range of these distributions was compared to those of the mutation effects in the near-neutral backgrounds using a Mann–Whitney *U* test.

### Relative mutation effect in non-wt backgrounds

Enrichment scores were computed for single and double mutants in the wt background. If we consider two single mutations A and B and their combination as a double mutation AB and their respective log2 enrichment score ES(A), ES(B) and ES(AB), the effect of A in the B background, ES(A->B)=ES(AB)−ES(B).

### Epistasis scores

We calculated the expected outcome when combining any two mutations by fitting a linear model to the relationship between the observed enrichment scores of all of the double mutants (ES(AB)), and the sums of the enrichments scores of the single mutants (ES(A)+ES(B)). This fit was performed separately on the data from each of the three replicate experiments. The epistasis score for each replicate is then the residual of the enrichment score of a double mutant from its expected score. The empirical epistasis score, *E*, for each double variant is the median of the residual scores for the three biological replicates. The significance of epistasis was calculated using a paired t-test between the three observed ES(AB) scores and the three ES(AB) scores predicted by the model. The *P*-values were corrected for multiple testing (false discovery rate, Benjamini–Hochberg procedure; interactions between near-neutral mutations (N × X)—*n*=5,110—and interactions between near-neutral mutations and any other mutation (N × X)—*n*=13,195). Interactions with a FDR<0.05 and an epistasis score<0 were classified as negative. Interactions with a FDR<0.05 and an epistasis score>0 were classified as positive. Otherwise, combinations were classified as non-epistatic. Epistasis scores and related information are listed in the Supplementary Data 3. The quantitative analysis of empirical epistasis scores presented in Supplementary Fig. 5 considered all mutations (X × X interactions; *n*=16,728).

### Data availability

Sequence data that support the findings of this study have been deposited in the European Nucleotide Archive with the primary accession code PRJEB13140.

## Additional information

**Accession codes:** Sequence data that support the findings of this study have been deposited in the European Nucleotide Archive with the primary accession code PRJEB13140.

**How to cite this article:** Julien, P. *et al*. The complete local genotype–phenotype landscape for the alternative splicing of a human exon. *Nat. Commun.* 7:11558 doi: 10.1038/ncomms11558 (2016).

## Supplementary Material

Supplementary Information Supplementary Figures 1-9 and Supplementary Table 1

Supplementary Data 1 Enrichment scores and information for single mutation variants. Enrichment scores, splicing effects categorization (S=increased skipping, I=increased inclusion, N=non-significant effect, X=nonsignificant effect with variance > 0.15), p-value (Welch's unequal variance t-test), FDR, ranking, standard deviation of replicates, median read coverage and the enrichment scores of all three replicates for all 189 single mutations variants. The ranks correspond to the ones used in Fig. 4E.

Supplementary Data 2 Enrichment scores of double mutation variants. Enrichment scores for all 16728 double mutant variants.

Supplementary Data 3 Epistasis score and information relative to variants' interactions. Epistasis scores and related information for 16728 combinations of single mutations.
