# Supplementary material for: The complete local genotype–phenotype landscape for the alternative splicing of a human exon
Source: Nat Commun. 2016 May 10;7:11558. doi: 10.1038/ncomms11558 (PMC4866304; doi:10.1038/ncomms11558)
Supplement: Supplementary Information — Supplementary Figures 1-9 and Supplementary Table 1 [file ncomms11558-s1.pdf]

Supplementary Information

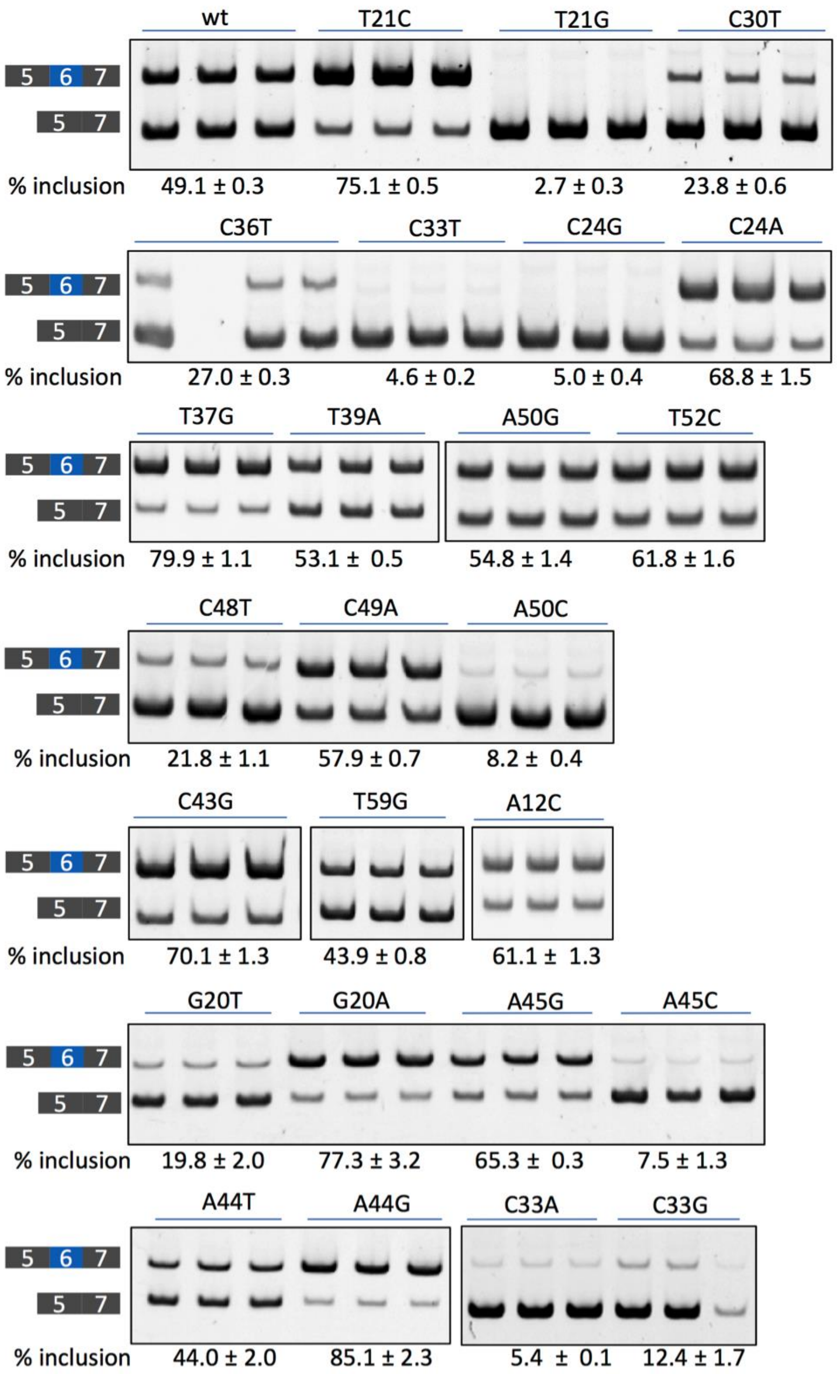

Supplementary Fig.1. Determination of inclusion rate for individually assayed single mutation variants in HEK293 cells. RT-PCR analysis of individually assayed single nucleotides variants, analysed as in Fig. 1D. Values represent average Percent Spliced In (PSI) and standard deviation for the three biological replicas presented for each mutant. Whole-gel images for these experiments are shown in Supplementary Fig. 9A.

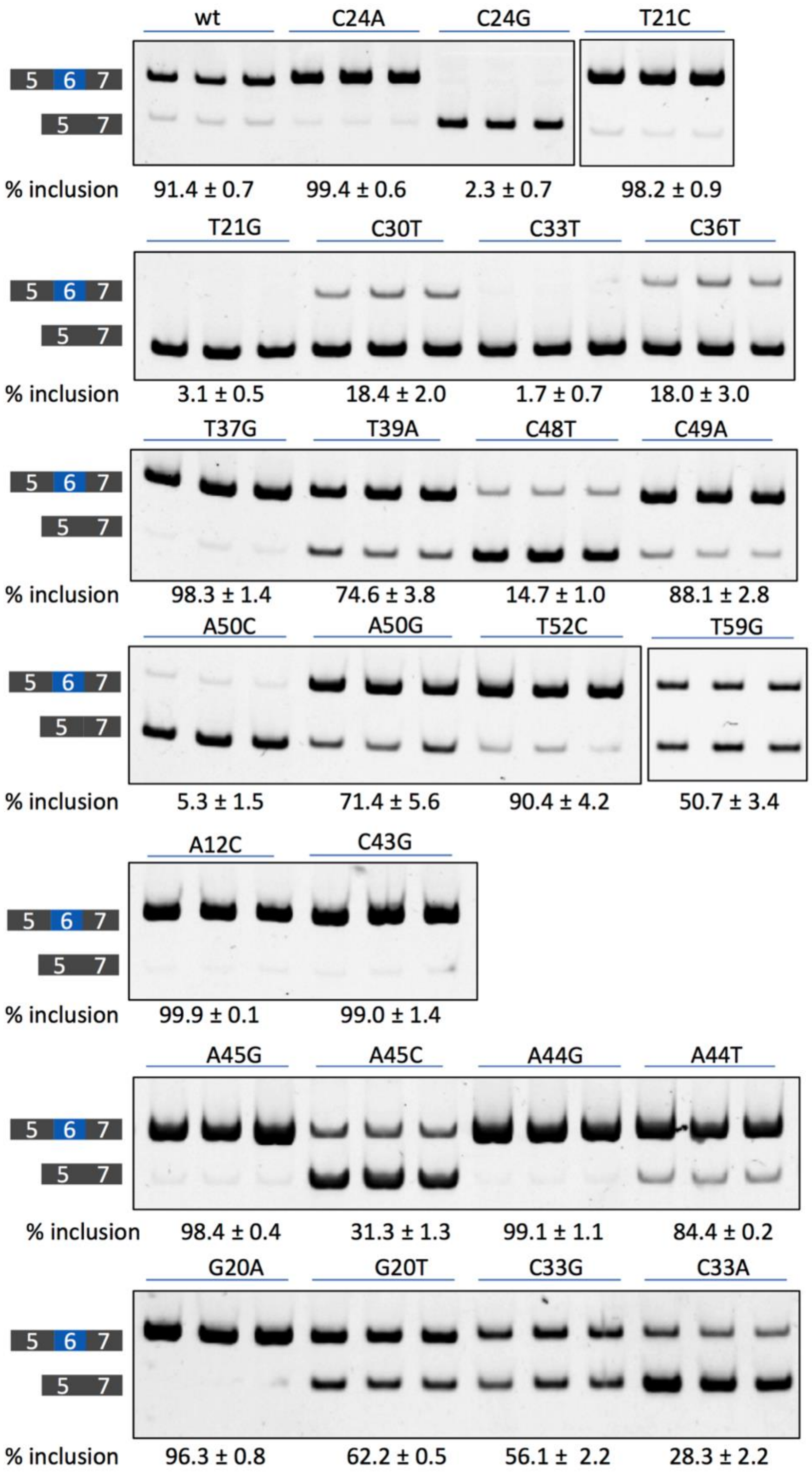

Supplementary Fig. 2. Determination of inclusion rate for individually assayed single mutation variants in HeLa cells. Whole-gel images for these experiments are shown in Supplementary Fig. 9B.

A

Sequence	Hek293	HeLa
FAS wt	49.1 ± 0.3	91.4 ± 0.7
T21C	75.1 ± 0.5	98.2 ± 0.9
T21G	2.7 ± 0.3	3.1 ± 0.5
C30T	23.8 ± 0.6	18.4 ± 2.0
C36T	27.0 ± 0.3	18.0 ± 3.0
C33T	4.6 ± 0.2	1.7 ± 0.7
C24G	5.0 ± 0.4	2.3 ± 0.7
C24A	68.8 ± 1.5	99.4 ± 0.6
T37G	79.9 ± 1.1	98.3 ± 1.4
T39A	53.1 ± 0.5	74.6 ± 3.8
A50G	54.8 ± 1.4	71.4 ± 5.6
T52C	61.8 ± 1.6	90.4 ± 4.2
C48T	21.8 ± 1.1	14.7 ± 1.0
C49A	57.9 ± 0.7	88.1 ± 2.8
A50C	8.2 ± 0.4	5.3 ± 1.5
C43G	70.1 ± 1.3	99.0 ± 1.4
T59G	43.9 ± 0.8	50.7 ± 3.4
A12C	61.1 ± 1.3	99.9 ± 0.1
G20T	19.8 ± 2.0	62.2 ± 0.5
G20A	77.3 ± 3.2	96.3 ± 0.8
A45G	65.3 ± 0.3	98.4 ± 0.4
A45C	7.5 ± 1.3	31.3 ± 1.3
A44T	44.0 ± 2.0	84.4 ± 0.2
A44G	85.1 ± 2.3	99.1 ± 1.1
C33A	5.4 ± 0.1	28.3 ± 2.2
C33G	12.4 ± 1.7	56.1 ± 2.2

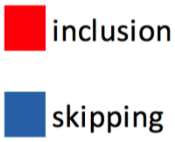

B

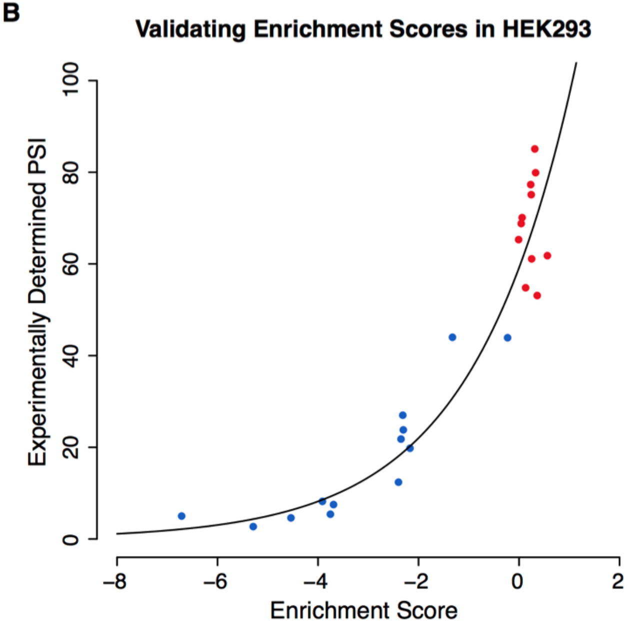

Supplementary Fig. 3. Inclusion levels in HEK293 and HeLa and the relationship between enrichment scores and inclusion levels.

A) Comparison of inclusion levels of individual mutants in HEK293 and HeLa cells. Numbers in red represent mutants whose inclusion levels are higher than those of the WT sequence. Numbers in blue come from mutants whose inclusion levels are lower than those of the WT sequence. B) To convert enrichment scores in HEK293 to percentage spliced in (PSI) values, we took advantage of the high correlation (r=0.96) between enrichment scores determined in transfection assays of individual mutant clones and by massively parallel RNA analysis of the transfected library (Figure 1C), to plot the enrichment scores of 24 single mutants in HEK293 against their experimentally-determined inclusion levels in HEK293. Red points are mutants whose inclusion levels are higher than the WT sequence, whereas blue points are mutants whose inclusion levels are lower than the WT sequence. The fitted curve shows the

relationship between enrichment scores and inclusion levels. Mutant C-49-A has an enrichment score of 1.46 and was not included in this graph as this was considered to be an outlier in the data.

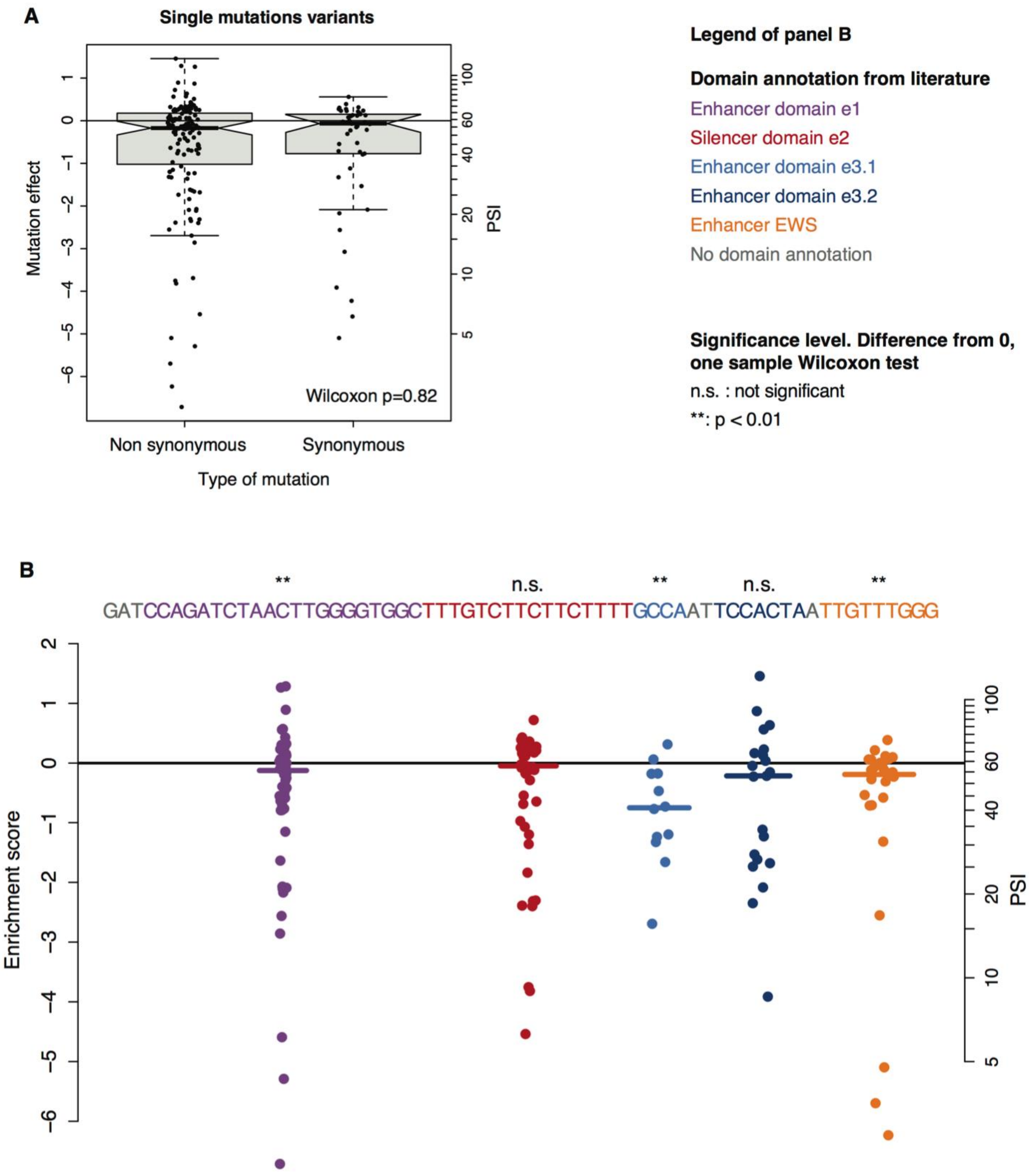

Supplementary Fig.4. Mutation effects of single nucleotide changes per mutation type and annotated domain. A) Enrichment scores of single mutations for synonymous and non-synonymous mutations. B) Enrichment scores of single mutation variants are visualized for regulatory domains previously reported in the literature [13, 14, 15]. 3/4 of the reported enhancer domains (in magenta, blue and orange) show a significantly higher skipping rate when

mutated, while the reported silencer domain (in red) shows no significant global change in splicing when collectively analysed through the effects of individual mutants.

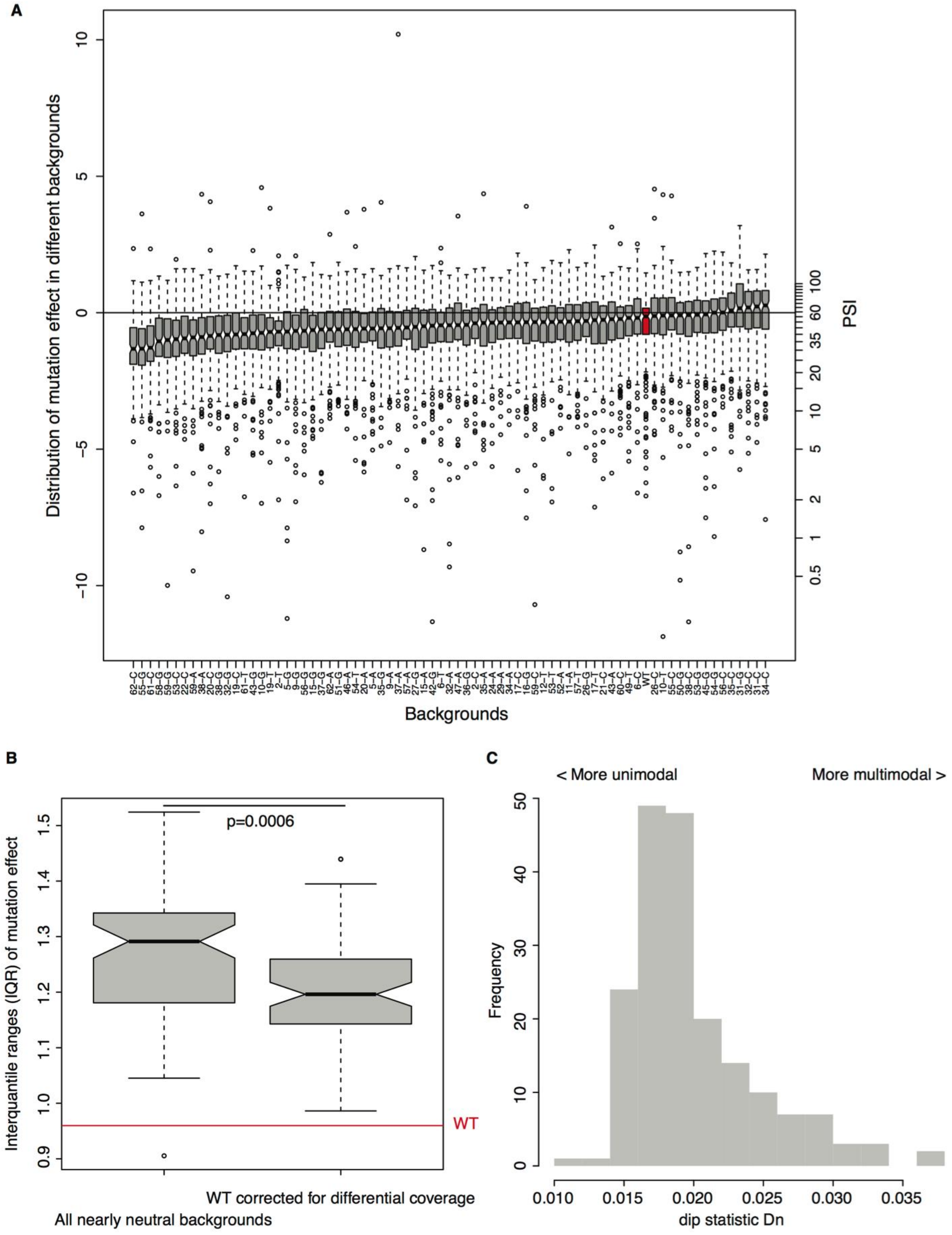

Supplementary Fig.5. Effect of single mutations in WT and near-neutral backgrounds. A) Mutation effects of all variants in the wild type (red) and in each of the 73 near-neutral

backgrounds (grey). B) Distribution breadth (as assessed by interquartile ranges; IQR) of the relative mutation effects of all variants in the 73 near-neutral backgrounds and relative mutation effect of all single mutations in 73 simulated WT backgrounds accounting for differential sequencing coverage. The horizontal red line represents the IQR of the non-corrected mutation effects in the wild type. C) Distribution of the dip Dn statistic for the relative effect of single mutations in all possible backgrounds. A low Dn value indicates a higher likeliness for the distribution to be unimodal. The distribution displays a long right tail, suggesting the presence of a subset of mutations with distinct effects in different backgrounds.

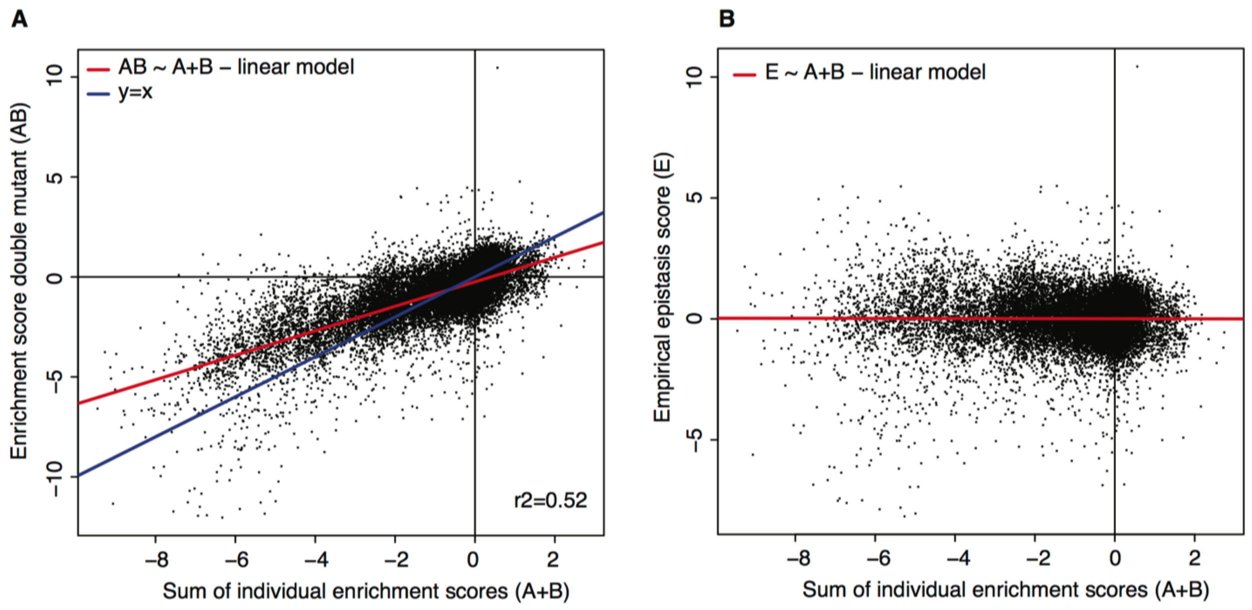

Supplementary Fig.6. Determination of empirical epistasis scores. A) Relation between enrichment scores of double mutants and the sum of the enrichment scores of its two single components. The red line represents the linear model best fitting the data, while the blue line represents the y=x line. B) Relation between empirical epistasis score (E) of an interaction and the sum of the enrichment scores of the two interactors (A+B). E is evenly distributed across different levels of A+B.

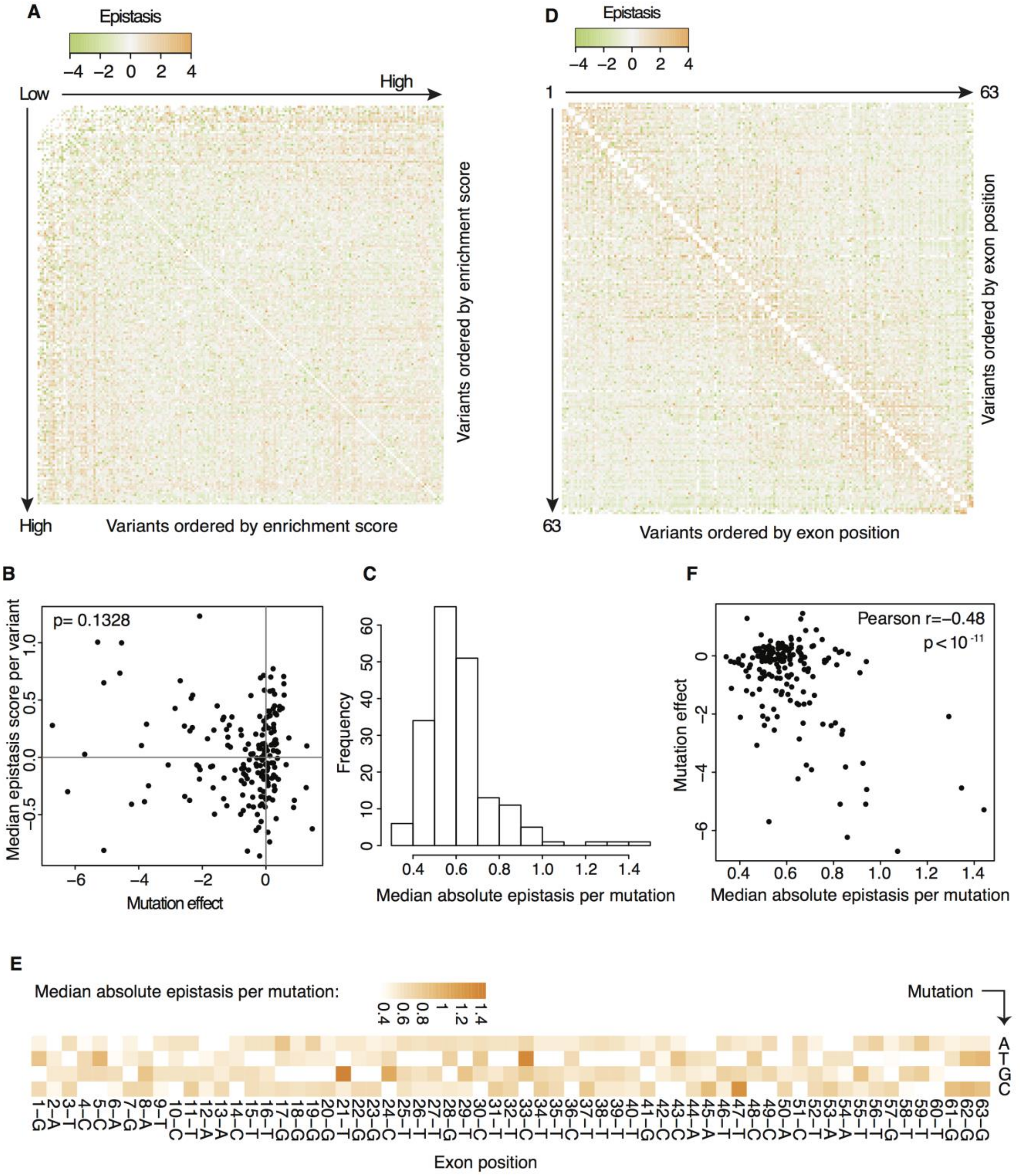

Supplementary Fig.7. Quantitative analysis of all possible pairwise mutation interactions. A) Heatmap displaying the empirical epistasis score for all 16728 interactions. Rows and columns represent the single variants and are ordered by those variants' scores. Combinations of low-inclusion variants are located on the top left corner. Values smaller or greater than -4 and +4 were set to -4 and +4 for better visualisation. B) Correlation between

single mutation variant scores and median epistasis score per single mutation variant. The correlation (pearson rho=-0.1097) is not significantly different from 0 (Fisher transformation p=0.1328). Altogether A) and B) show that there is no global and significant bias of empirical epistasis scores across different levels of inclusion scores. C) Distribution of median absolute epistasis per position. The long right tail indicates potential hubs, mutations often interacting epistatically with other mutations. D) Heatmap visualising the epistasis values for all interactions. Rows and columns represent the single variants and are ordered by the position of the mutated site in the exon. Values smaller or greater than -4 and +4 were set to -4 and +4 for better visualisation. Positively epistatic interactions are enriched for local interactions (diagonal) E) Median absolute epistasis per position displayed for each possible substitutions. Epistasis hubs correspond to distinct exon positions. F) Correlation between the median absolute epistasis per substitution and the inclusion score in the wt sequence. Epistasis hubs are more likely to be, but are not necessarily, variants with low inclusion rate in the wt sequence.

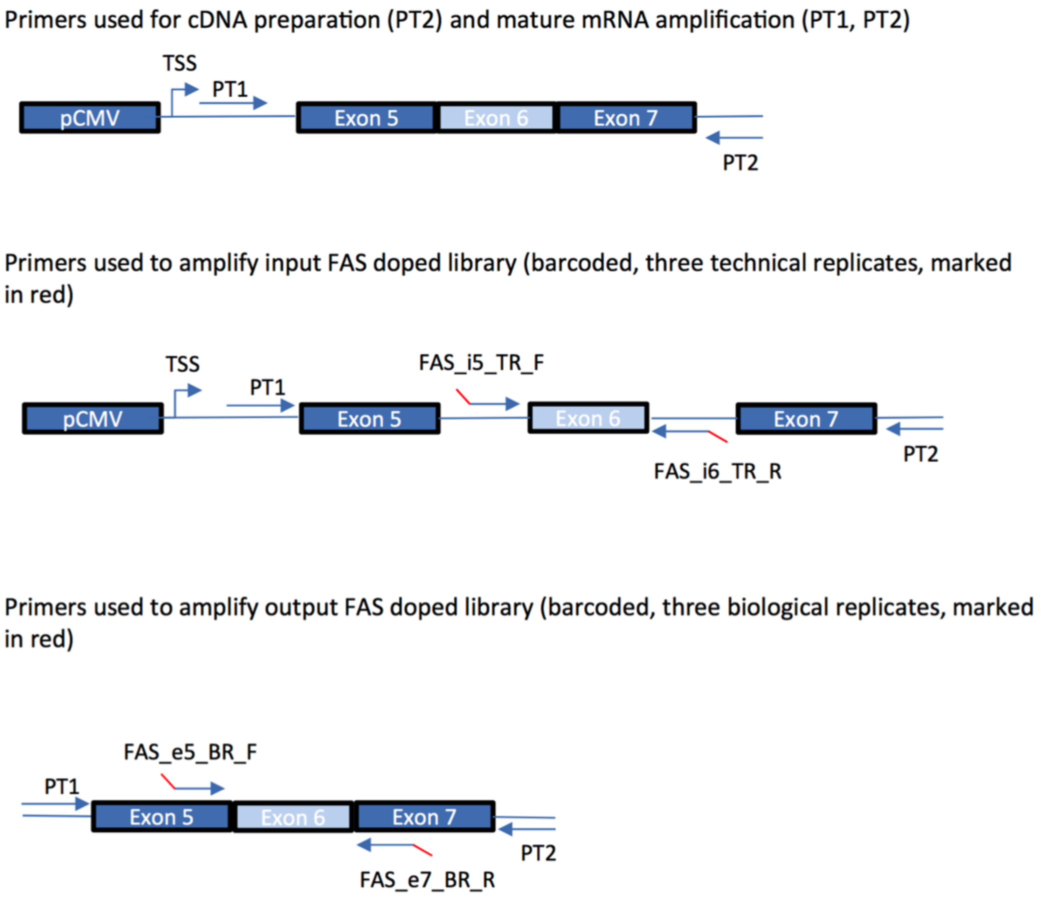

Supplementary Fig. 8. Schematic representation of primers used for cDNA synthesis, input library barcoding and output library barcoding. Primer sequences are provided in Supplementary Table 1.

Supplementary Fig. 9. Whole gels for experiments shown in Fig.1, Supplementary Fig.1 and Supplementary Fig.2. In all the gels shown, the molecular-weight size marker used was a Thermo Scientific GeneRuler 50 bp DNA ladder. A) Experiments carried out in HEK293 cells, corresponding to Fig. 1D and Supplementary Fig.1. B) Experiments carried out in HeLa cells, corresponding to Supplementary Fig. 2.

A. HEK293

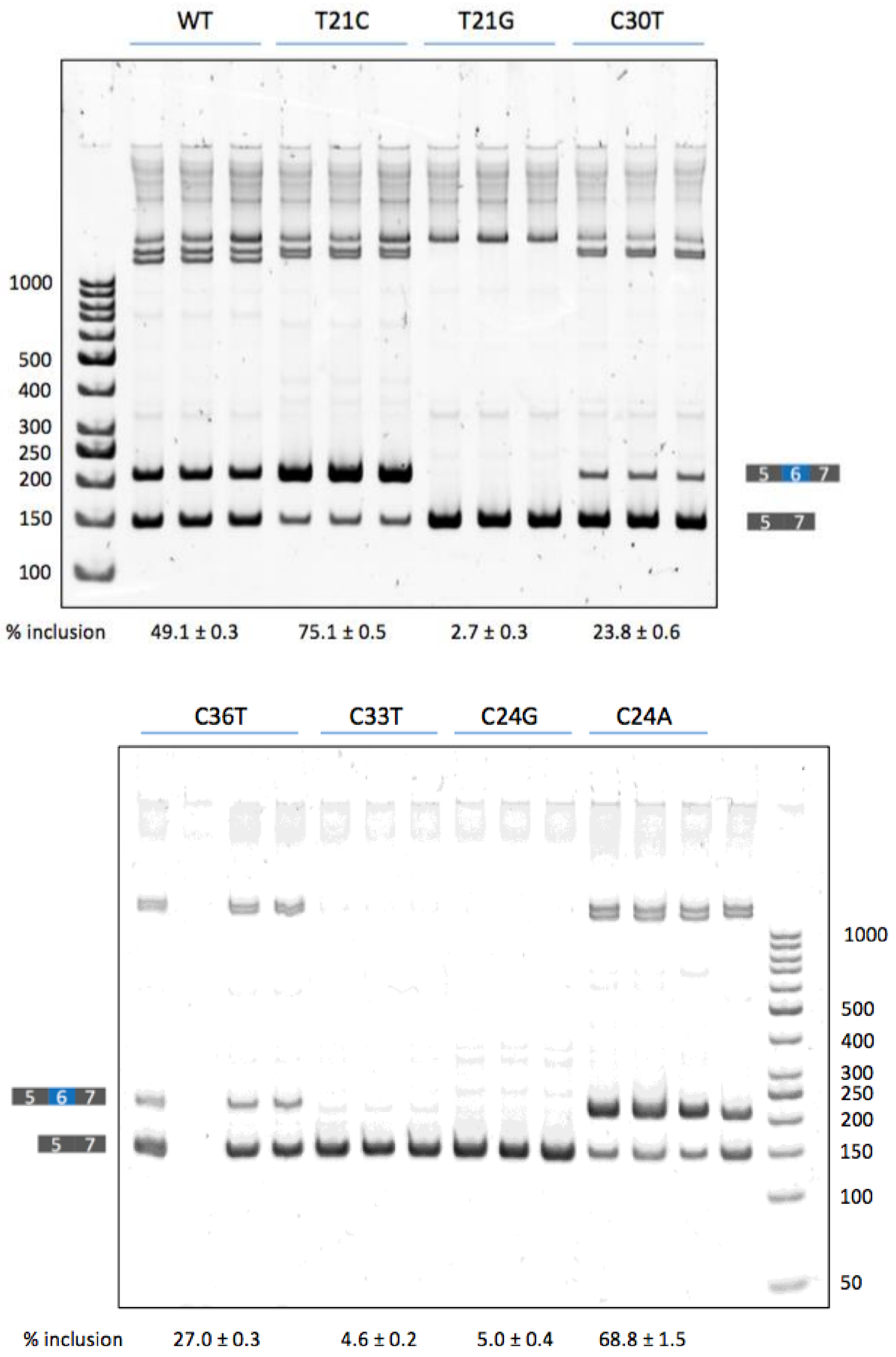

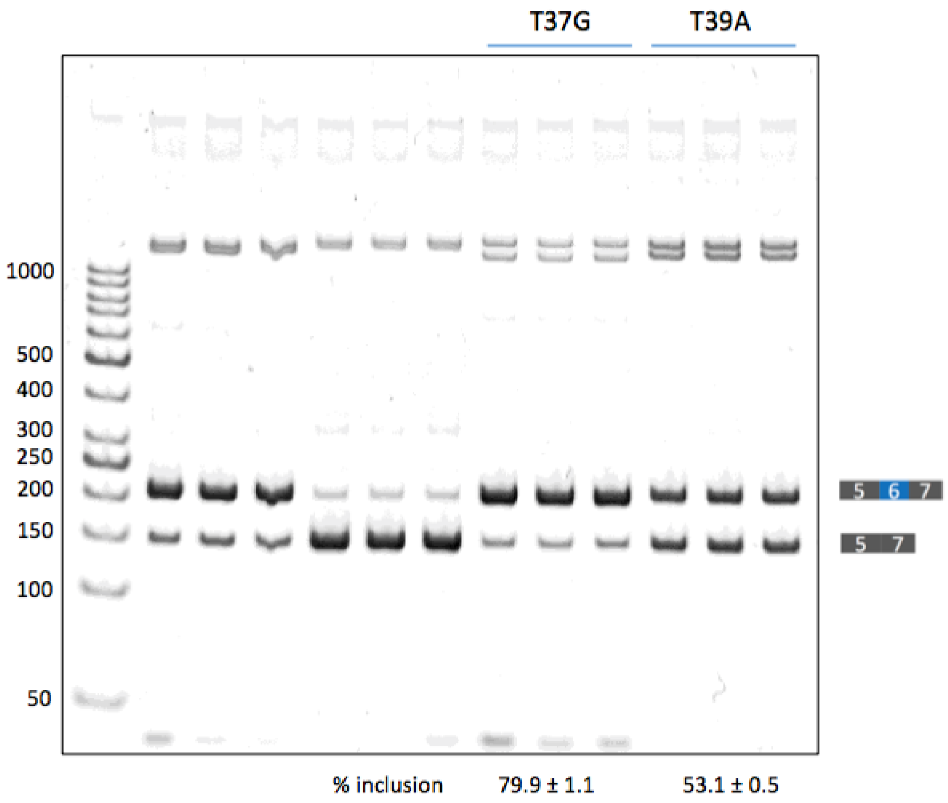
T37G
T39A
1000
500
400
300
250
200
150
100
50
5 6 7
5 7
% inclusion
79.9 ± 1.1
53.1 ± 0.5

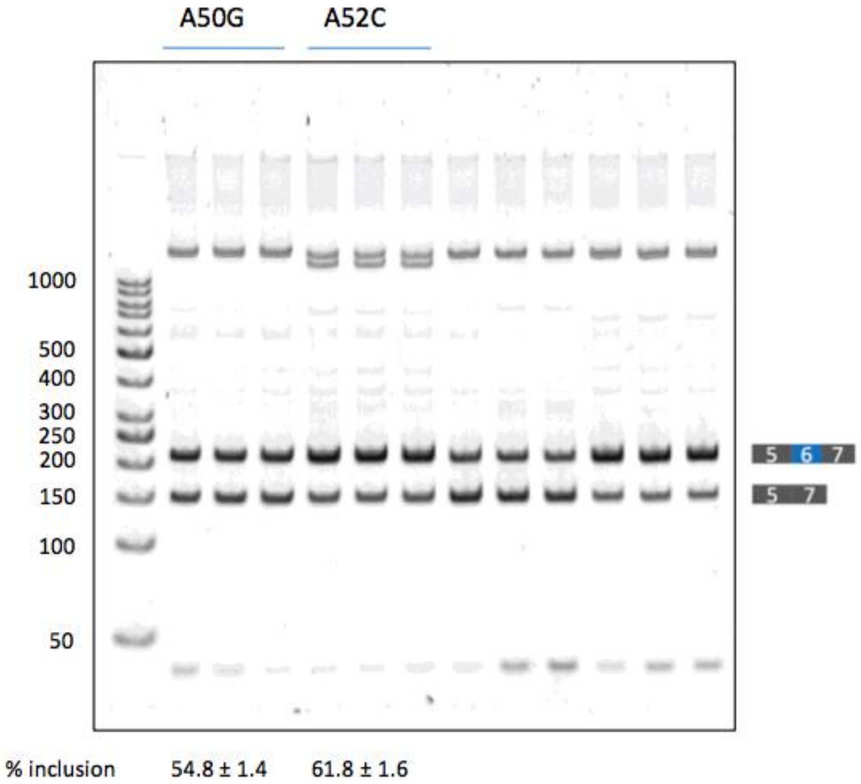
A50G
A52C
1000
500
400
300
250
200
150
100
50
5 6 7
5 7
% inclusion
54.8 ± 1.4
61.8 ± 1.6

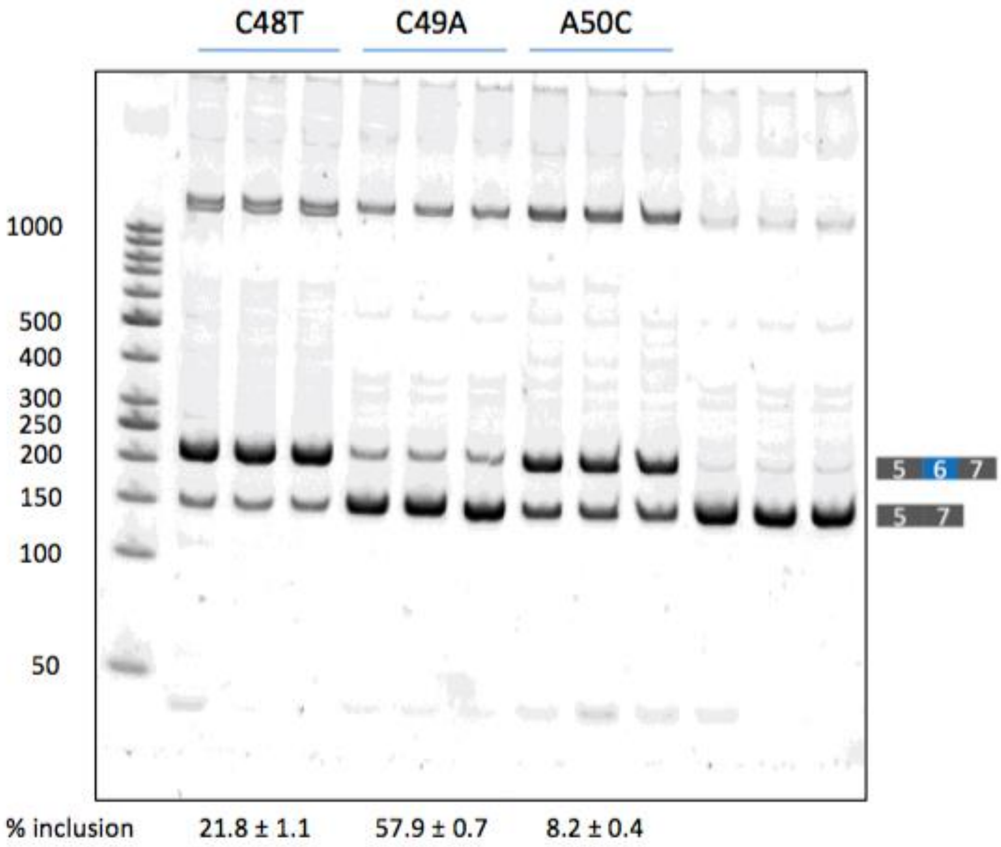
C48T
C49A
A50C
1000
500
400
300
250
200
150
100
50
5 6 7
5 7
% inclusion
21.8 ± 1.1
57.9 ± 0.7
8.2 ± 0.4

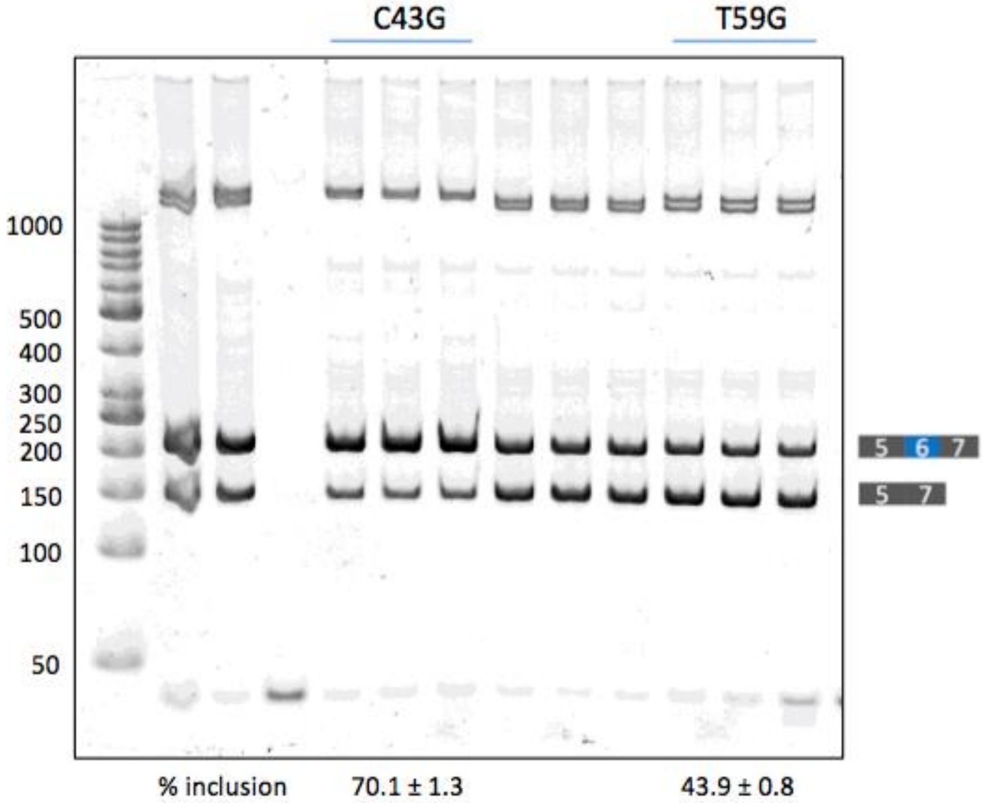
C43G
T59G
1000
500
400
300
250
200
150
100
50
5 6 7
5 7
% inclusion
70.1 ± 1.3
43.9 ± 0.8

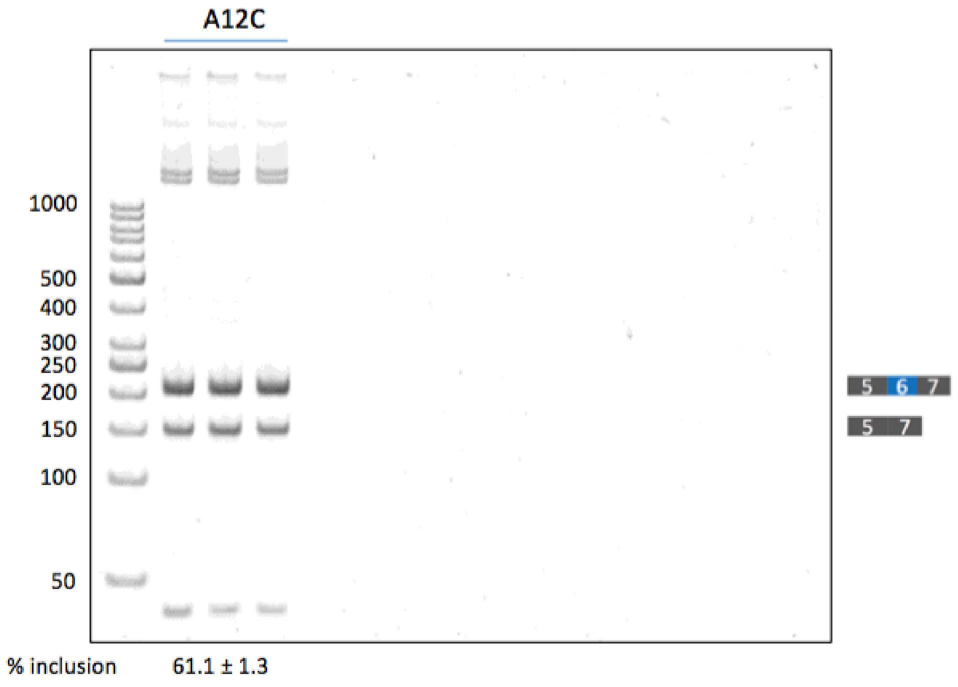
A12C
1000
500
400
300
250
200
150
100
50
5 6 7
5 7
% inclusion
61.1 ± 1.3

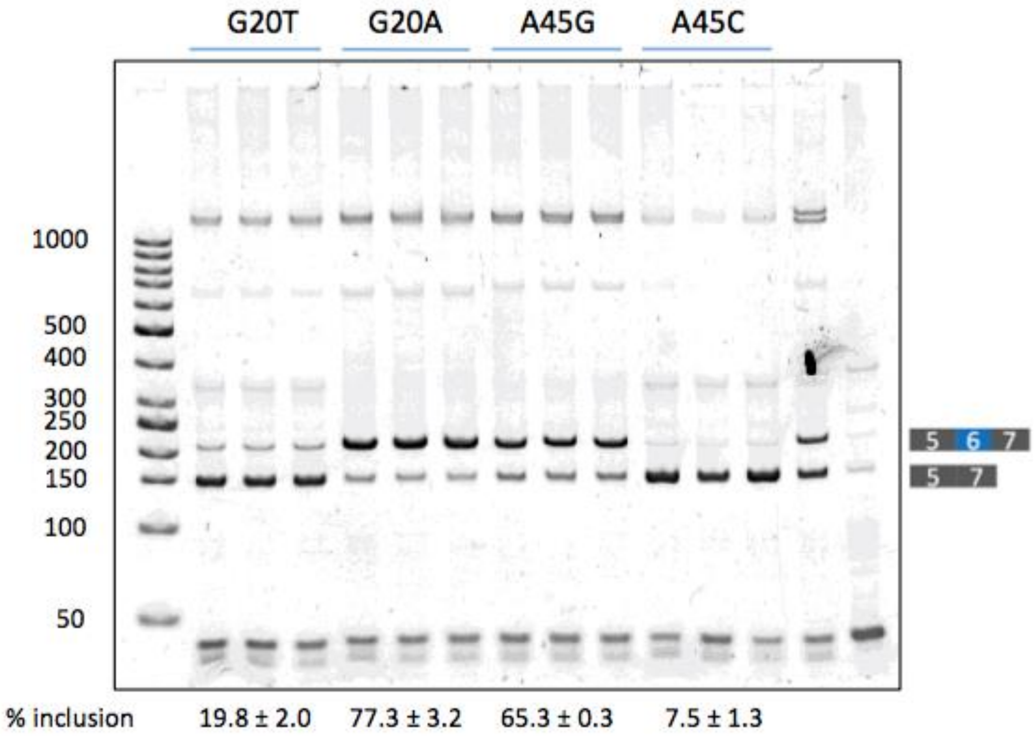
G20T
G20A
A45G
A45C
1000
500
400
300
250
200
150
100
50
5 6 7
5 7
% inclusion
19.8 ± 2.0
77.3 ± 3.2
65.3 ± 0.3
7.5 ± 1.3

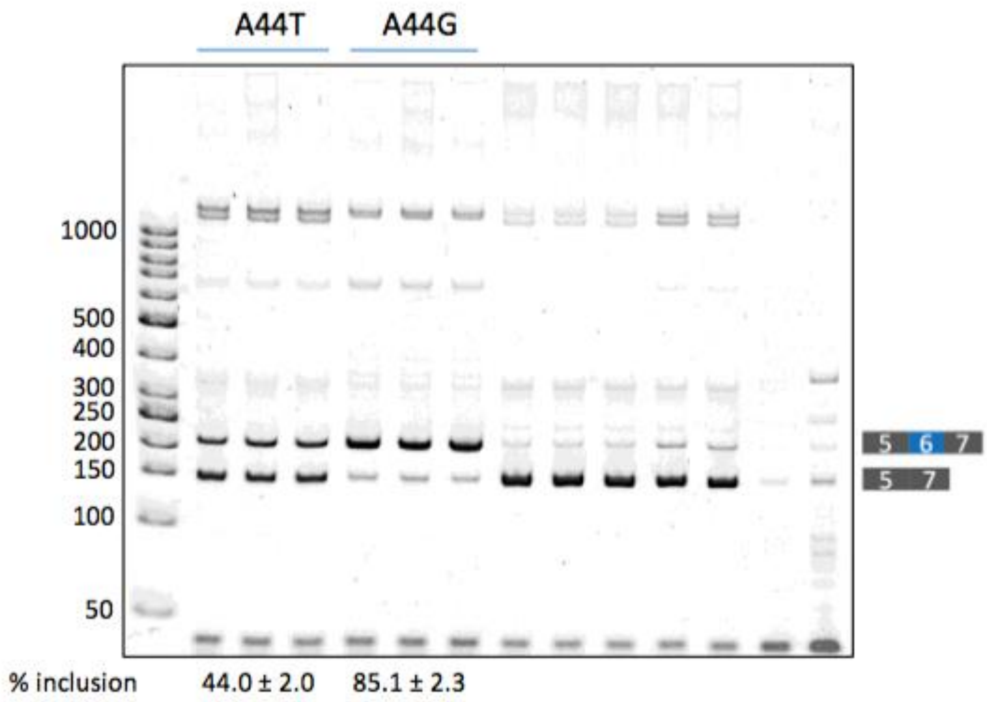
A44T
A44G
1000
500
400
300
250
200
150
100
50
5 6 7
5 7
% inclusion
44.0 ± 2.0
85.1 ± 2.3

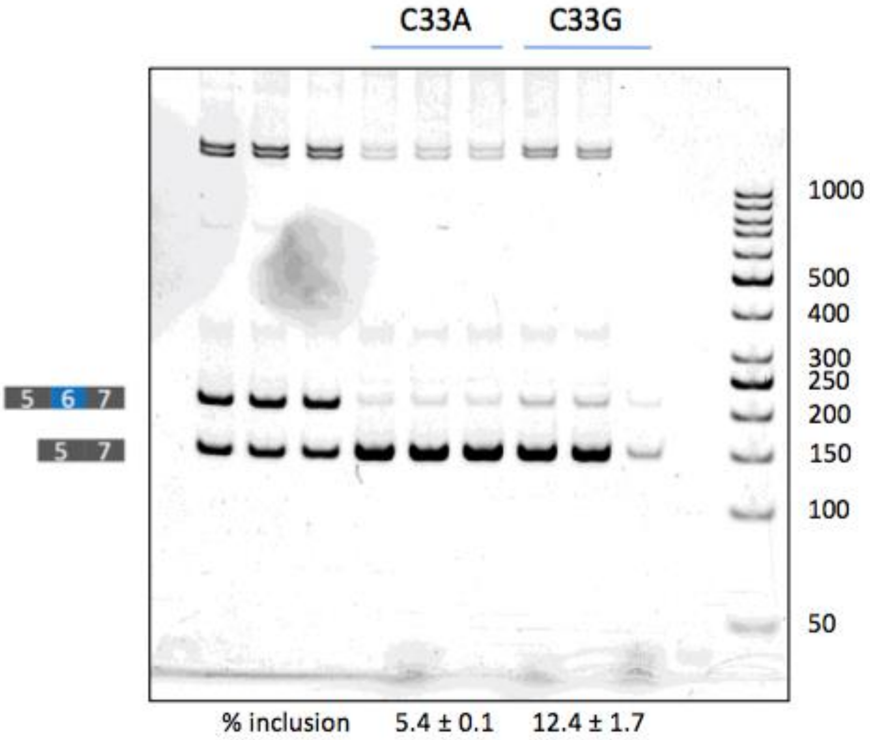
C33A
C33G
1000
500
400
300
250
200
150
100
50
5 6 7
5 7
% inclusion
5.4 ± 0.1
12.4 ± 1.7

B. HeLa

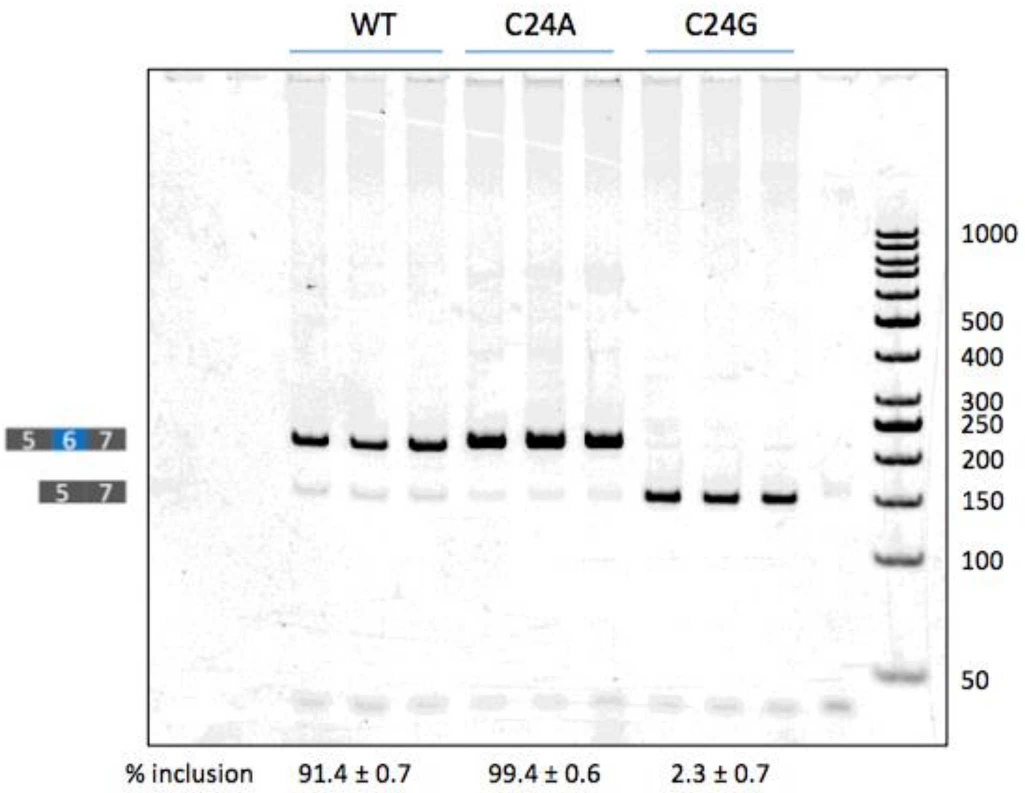
WT
C24A
C24G
5 6 7
5 7
1000
500
400
300
250
200
150
100
50
% inclusion
91.4 ± 0.7
99.4 ± 0.6
2.3 ± 0.7

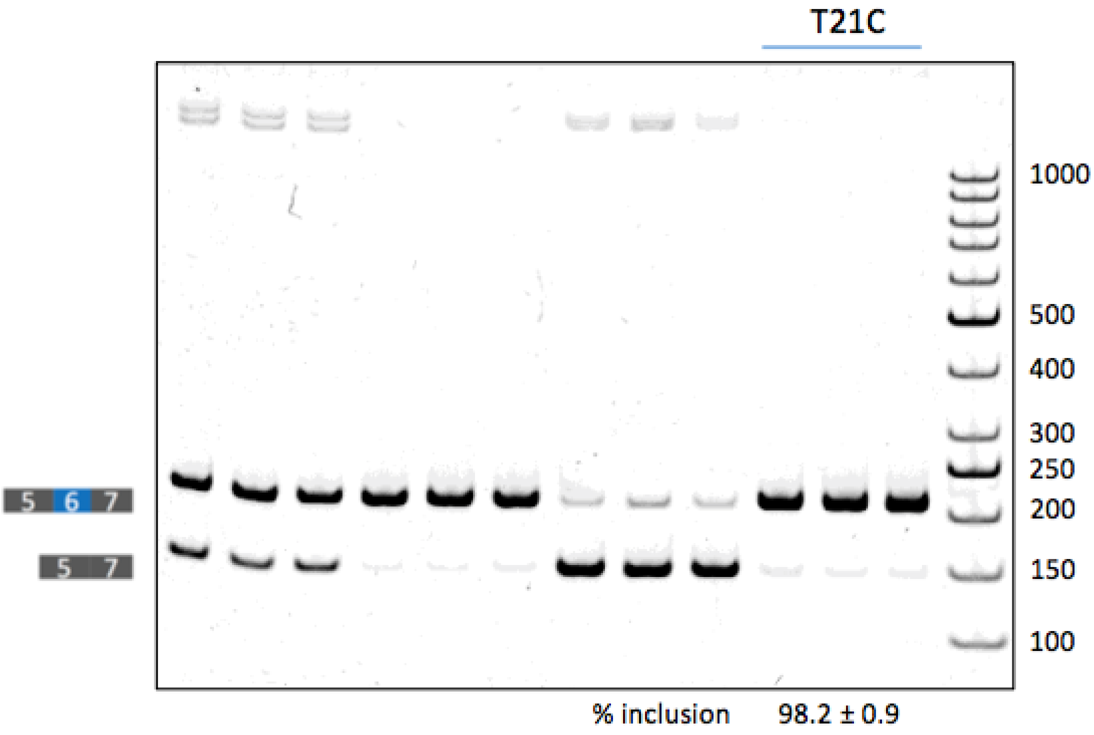
T21C
5 6 7
5 7
1000
500
400
300
250
200
150
100
% inclusion
98.2 ± 0.9

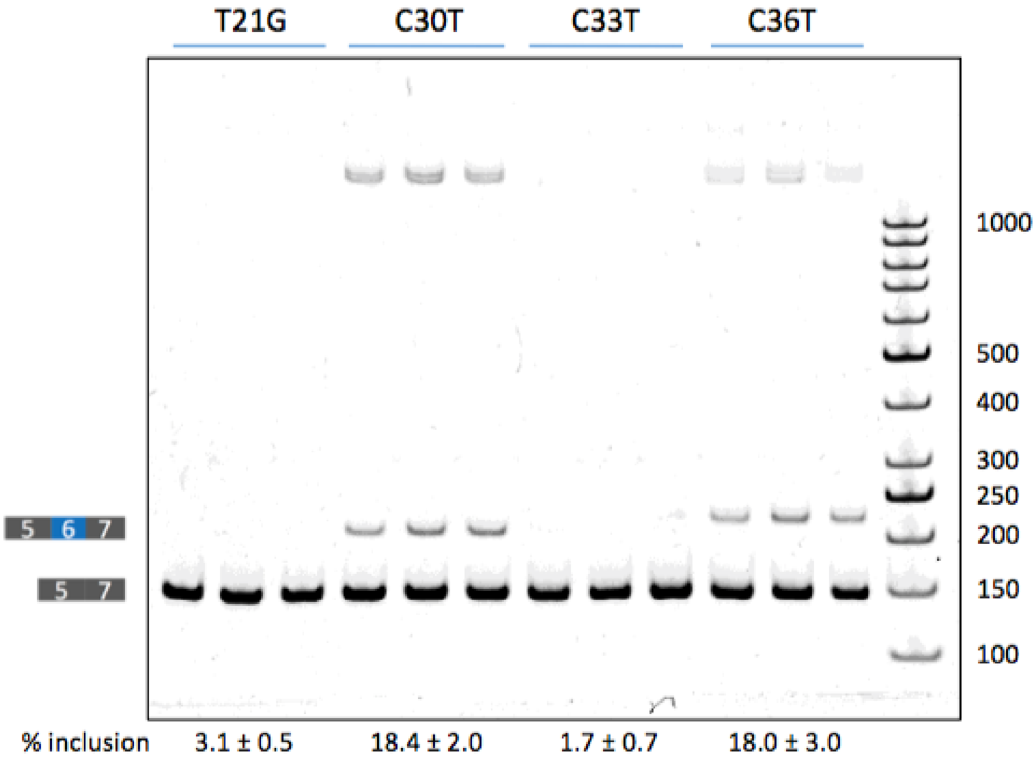
T21G
C30T
C33T
C36T
1000
500
400
300
250
200
150
100
5 6 7
5 7
% inclusion
3.1 ± 0.5
18.4 ± 2.0
1.7 ± 0.7
18.0 ± 3.0

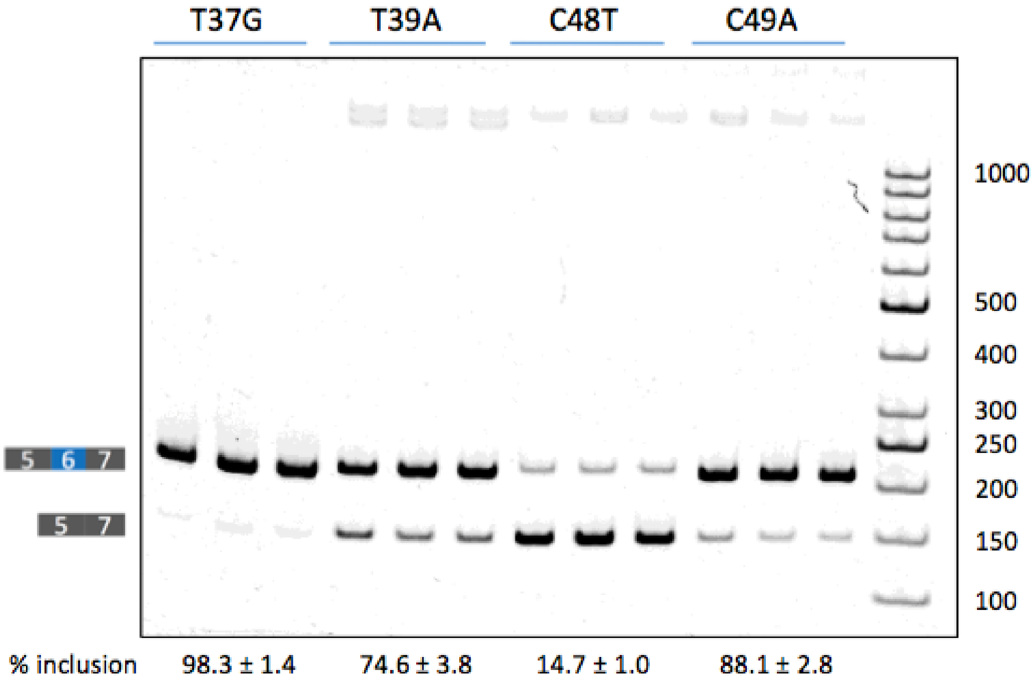
T37G
T39A
C48T
C49A
1000
500
400
300
250
200
150
100
5 6 7
5 7
% inclusion
98.3 ± 1.4
74.6 ± 3.8
14.7 ± 1.0
88.1 ± 2.8

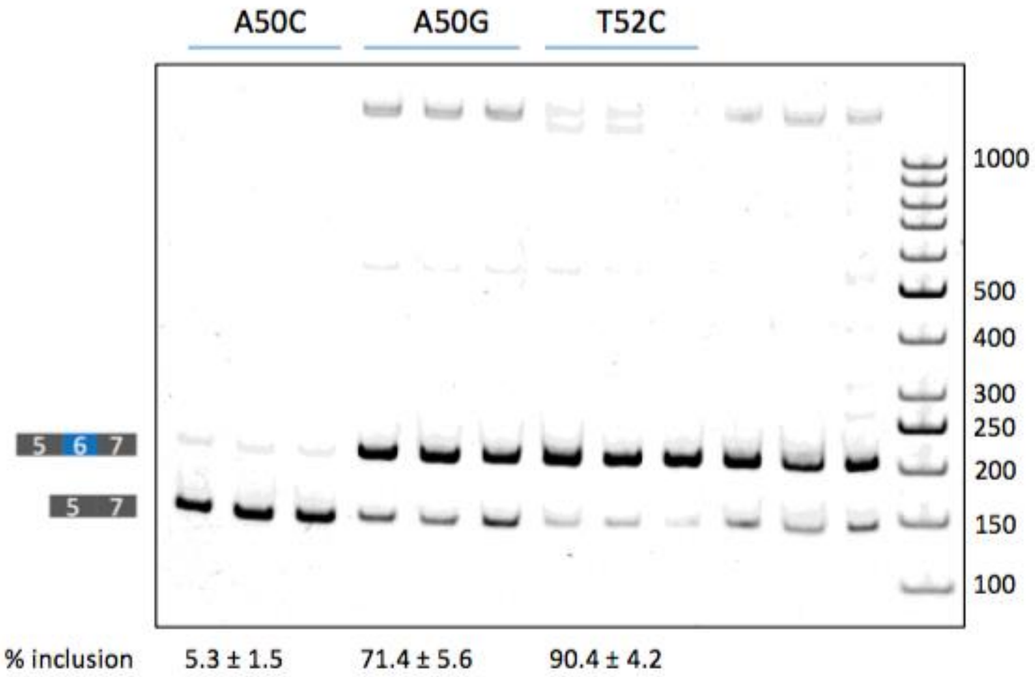

A50C
A50G
T52C
1000
500
400
300
250
200
150
100
5 6 7
5 7
% inclusion
5.3 ± 1.5
71.4 ± 5.6
90.4 ± 4.2

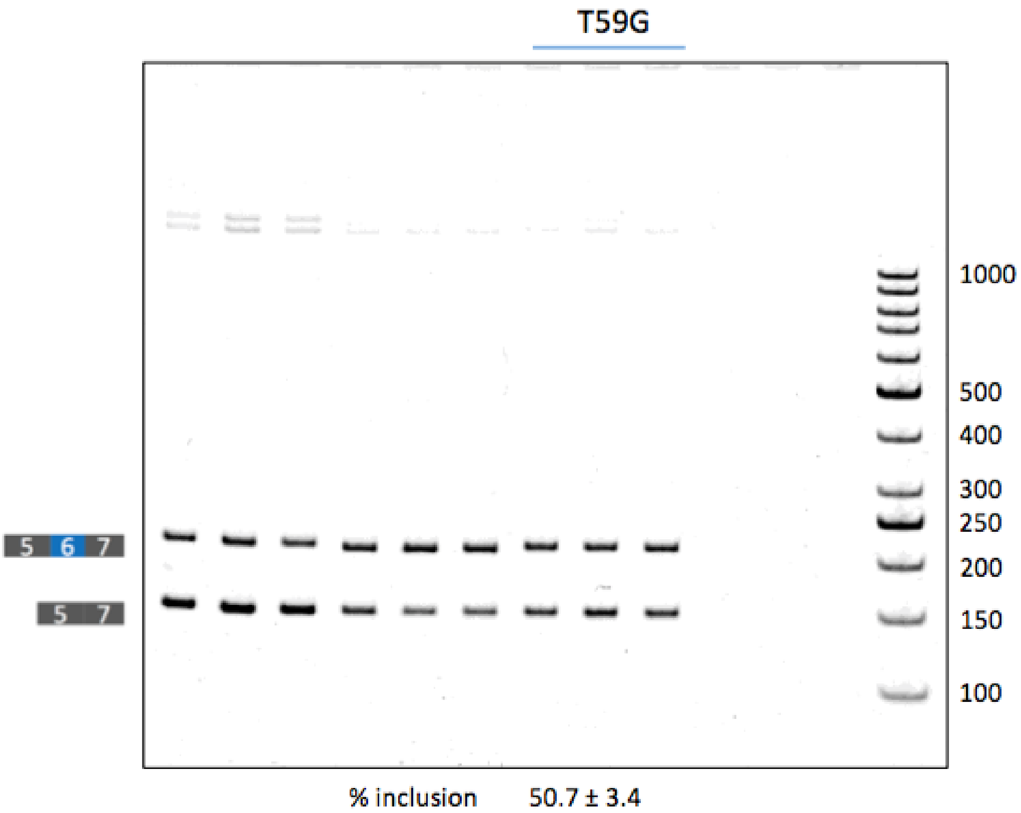

T59G
1000
500
400
300
250
200
150
100
5 6 7
5 7
% inclusion
50.7 ± 3.4

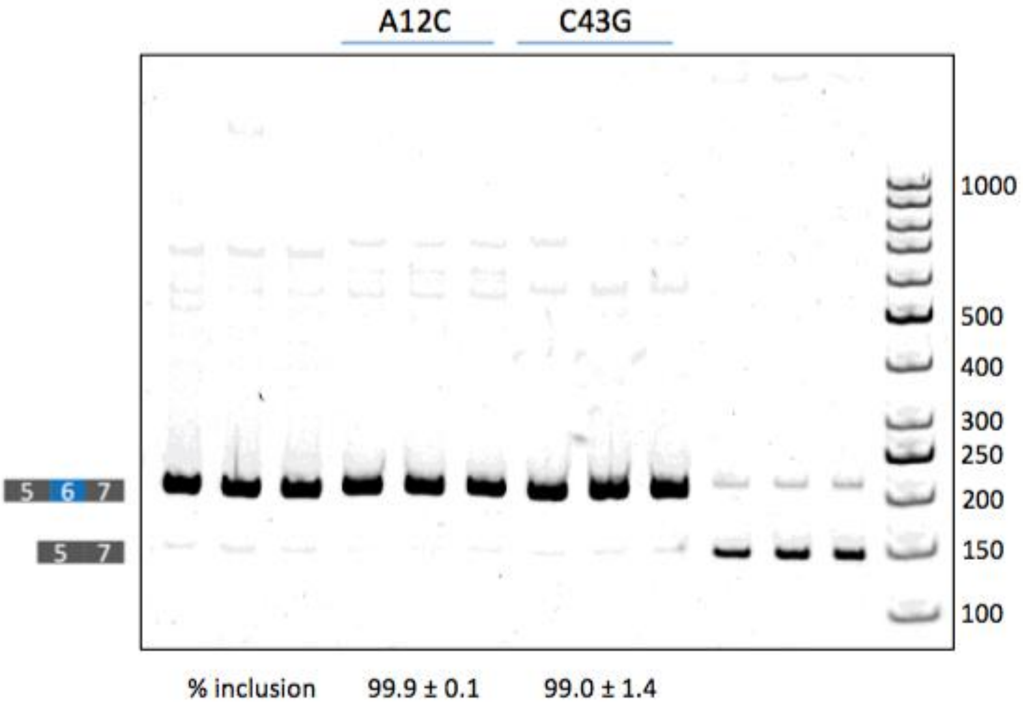
A12C
C43G
1000
500
400
300
250
200
150
100
5 6 7
5 7
% inclusion
99.9 ± 0.1
99.0 ± 1.4

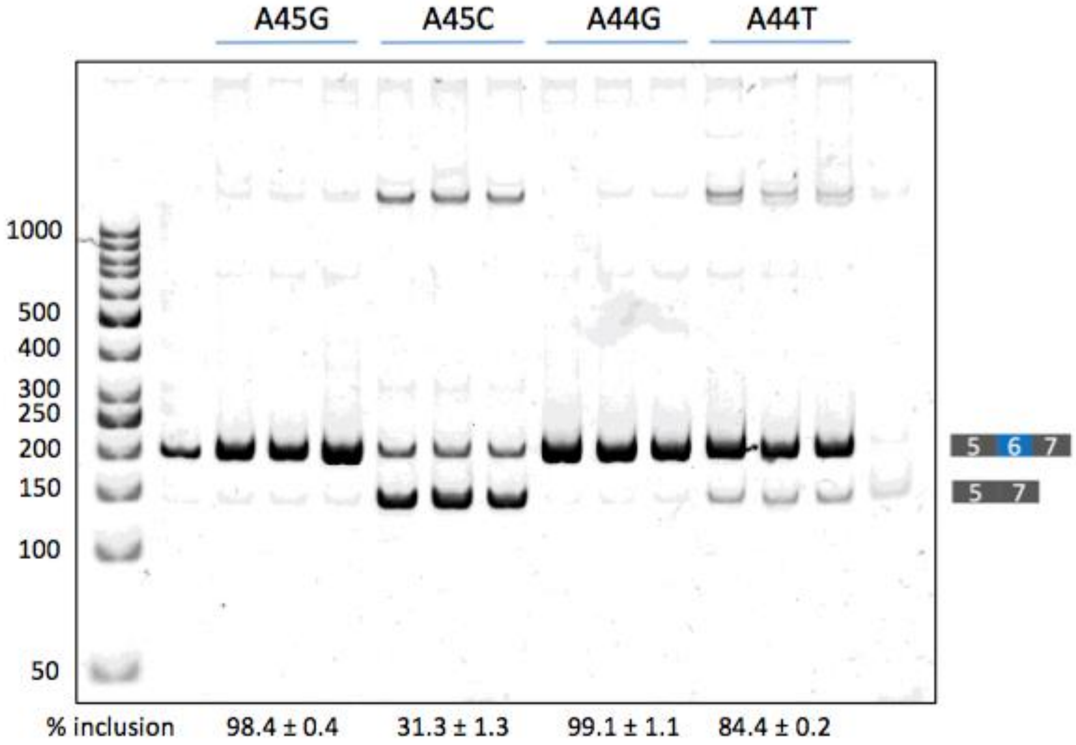
A45G
A45C
A44G
A44T
1000
500
400
300
250
200
150
100
50
5 6 7
5 7
% inclusion
98.4 ± 0.4
31.3 ± 1.3
99.1 ± 1.1
84.4 ± 0.2

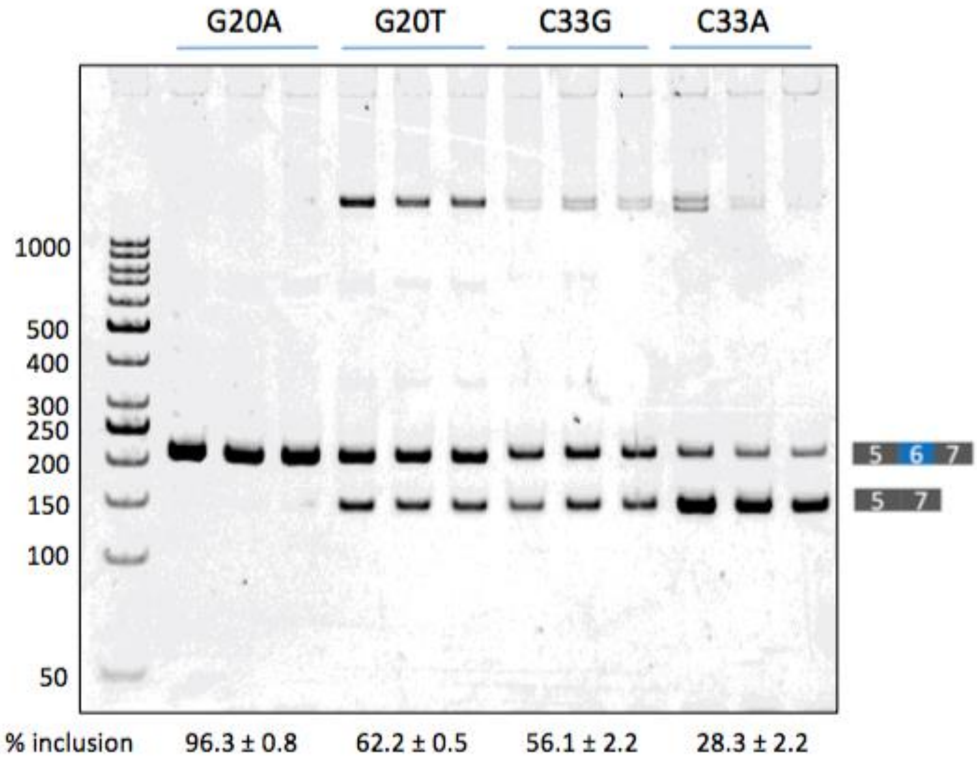
G20A
G20T
C33G
C33A
1000
500
400
300
250
200
150
100
50
5 6 7
5 7
% inclusion
96.3 ± 0.8
62.2 ± 0.5
56.1 ± 2.2
28.3 ± 2.2

Supplementary Table

Barcoded primers used for Ampliseq sequencing	Replicate	Replicate	Forward primers	BARCODE	Forward primer sequence	Reverse primers	BARCODE	Reverse primer sequence
output library	Biological	1	FAS_e5_BR_F1	GCCGAATT	**CAGCAACACCAAGTGCAAAG**	FAS_e7_BR_R1	GCCGAATT	**TGCATGTTTTCTGTACTTCCTTTC**
output library	Biological	2	FAS_e5_BR_F2	CGGCAATT	**CAGCAACACCAAGTGCAAAG**	FAS_e7_BR_R2	CGGCAATT	**TGCATGTTTTCTGTACTTCCTTTC**
output library	Biological	3	FAS_e5_BR_F3	GAACGTTC	**CAGCAACACCAAGTGCAAAG**	FAS_e7_BR_R3	GAACGTTC	**TGCATGTTTTCTGTACTTCCTTTC**
input library	Technical	1	FAS_i5_TR_F1	GCCGAATT	**aaaatgtccaatgttccaacc**	FAS_i6_TR_R1	GCCGAATT	**tgcagtttgaacaaagcaaga**
input library	Technical	2	FAS_i5_TR_F2	CGGCAATT	**aaaatgtccaatgttccaacc**	FAS_i6_TR_R2	CGGCAATT	**tgcagtttgaacaaagcaaga**
input library	Technical	3	FAS_i5_TR_F3	GAACGTTC	**aaaatgtccaatgttccaacc**	FAS_i6_TR_R3	GAACGTTC	**tgcagtttgaacaaagcaaga**

Primers used for RT-PCR	Sequence
PT1	GTCGACGACACTTGCTCAAC
PT2	AAGCTTGCATCGAATCAGTAG

Primers used for doped library amplification	Sequence
FAS_i5_GC_F	tgtccaatgttccaacctacag
FAS_i6_GC_R	ctacttcccaagttatttcaatctg

Supplementary Table 1. Primer sequences for cDNA synthesis, input library barcoding and output library and barcode information for multiplexed sequencing libraries. Barcodes associated with each sample in the sequencing libraries are indicated. The three input replicates were pooled and sequenced in one lane. The three output replicates were pooled and sequenced in another lane.
